# Diagnostics for a troubled backbone: testing topological hypotheses of trapelioid lichenized fungi in a large-scale phylogeny of Ostropomycetidae (Lecanoromycetes)

**DOI:** 10.1007/s13225-015-0332-y

**Published:** 2015-05-13

**Authors:** Philipp Resl, Kevin Schneider, Martin Westberg, Christian Printzen, Zdeněk Palice, Göran Thor, Alan Fryday, Helmut Mayrhofer, Toby Spribille

**Affiliations:** Institute of Plant Sciences, NAWI Graz, University of Graz, Holteigasse 6, A-8010 Graz, Austria; Department of Botany, Swedish Museum of Natural History, P.O. Box 50007, SE-104 05 Stockholm, Sweden; Senckenberg Forschungsinstitut und Naturmuseum, Senckenberganlage 25, D-60325 Frankfurt am Main, Germany; Institute of Botany, Academy of Sciences of the Czech Republic, Zámek 1, 252 43 Průhonice, Czech Republic; Department of Botany, Faculty of Sciences, Charles University in Prague, Benátská 2, 128 01 Praha 2, Czech Republic; Department of Ecology, Swedish University of Agricultural Sciences, P. O. Box 7044, SE-750 07 Uppsala, Sweden; Herbarium, Department of Plant Biology, Michigan State University, East Lansing, MI 48824 USA; Division of Biological Sciences, University of Montana, 32 Campus Drive, Missoula, MT 59812 USA

**Keywords:** Ascomycota, Fungi, *Lambiella*, Lecanoromycetes, Ostropomycetidae, *Parainoa*, Paraphyly, SOWH test, Taxon sampling

## Abstract

Trapelioid fungi constitute a widespread group of mostly crust-forming lichen mycobionts that are key to understanding the early evolutionary splits in the Ostropomycetidae, the second-most species-rich subclass of lichenized Ascomycota. The uncertain phylogenetic resolution of the approximately 170 species referred to this group contributes to a poorly resolved backbone for the entire subclass. Based on a data set including 657 newly generated sequences from four ribosomal and four protein-coding gene loci, we tested a series of a priori and new evolutionary hypotheses regarding the relationships of trapelioid clades within Ostropomycetidae. We found strong support for a monophyletic group of nine core trapelioid genera but no statistical support to reject the long-standing hypothesis that trapelioid genera are sister to Baeomycetaceae or Hymeneliaceae. However, we can reject a sister group relationship to Ostropales with high confidence. Our data also shed light on several long-standing questions, recovering Anamylopsoraceae nested within Baeomycetaceae, elucidating two major monophyletic groups within trapelioids (recognized here as Trapeliaceae and Xylographaceae), and rejecting the monophyly of the genus *Rimularia*. We transfer eleven species of the latter genus to *Lambiella* and describe the genus *Parainoa* to accommodate a previously misunderstood species of *Trapeliopsis*. Past phylogenetic studies in Ostropomycetidae have invoked “divergence order” for drawing taxonomic conclusions on higher level taxa. Our data show that if backbone support is lacking, contrasting solutions may be recovered with different or added data. We accordingly urge caution in concluding evolutionary relationships from unresolved phylogenies.

**Electronic supplementary material:**

The online version of this article (doi:10.1007/s13225-015-0332-y) contains supplementary material, which is available to authorized users.

## Introduction

**Fig. 1:**
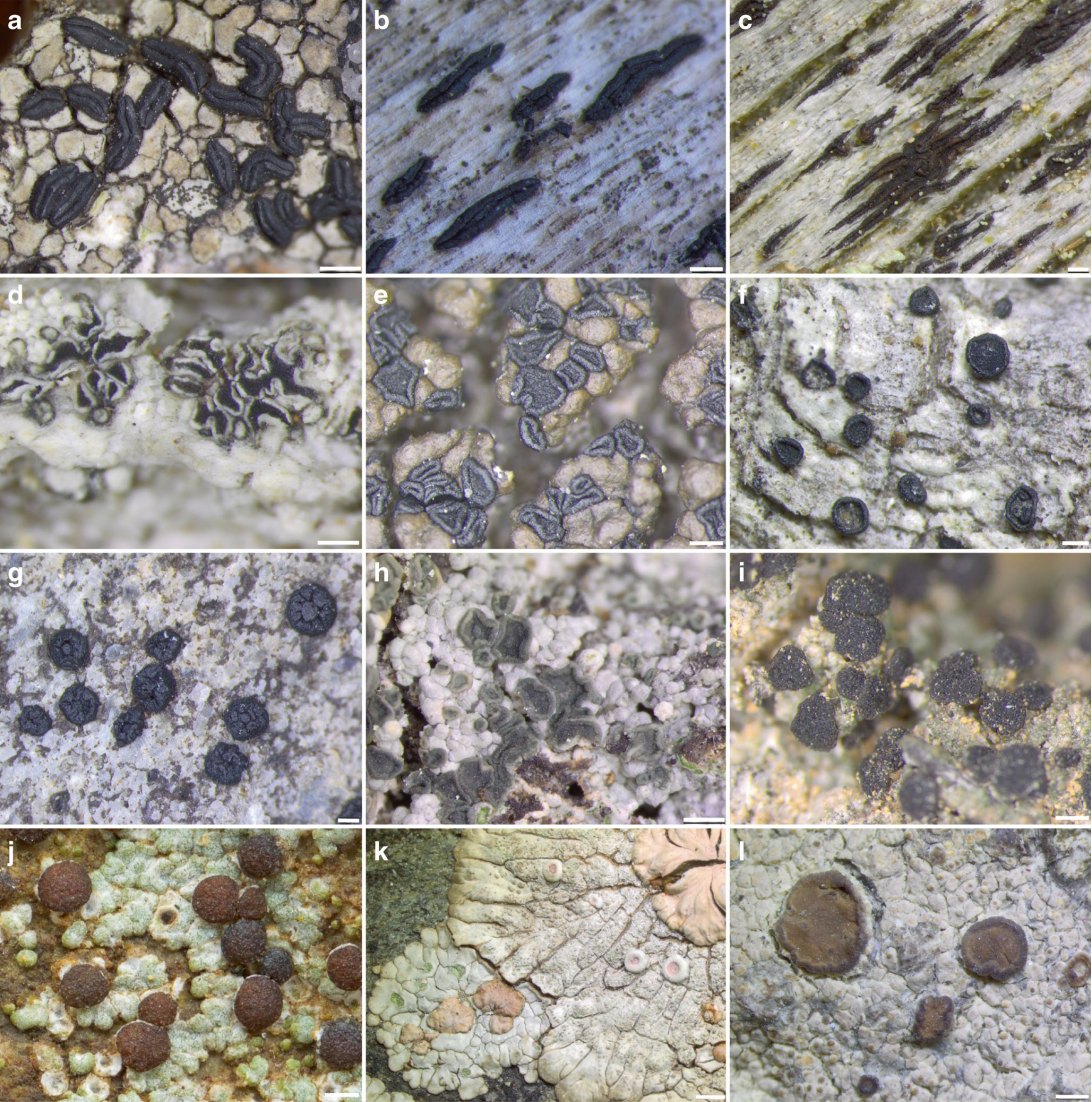
Diversity of trapelioid fungi in the broad sense, grouped by the major clades recovered here. **a** to **f**, **Xylographaceae. a**, *Lithographa tesserata* (Alaska, Spribille [=S] 38950, GZU; scale bar 0.5 mm); **b**, *Ptychographa xylographoides* (Scotland, Coppins 24229, GZU: 200 μm); **c**, *Xylographa pallens* (Austria, Resl 1143, GZU: 200 μm); **d**, *Xylographa lagoi* (Spain, S30267, GZU: 200 μm); **e**, *Lambiella insularis* (Montana, S/07.09.2012, GZU: 200 μm); **f**, *Lambiella caeca* (Alaska, S36295, GZU: 200 μm); **g** to **k**, **Trapeliaceae. g**, *Rimularia limborina* (Alaska, Fryday 10100, MSC: 200 μm); **h**, *Trapeliopsis granulosa* (Sweden, Nordin 7402, UPS: 500 μm); **i**, *Placynthiella uliginosa* (Montana, S/21.09.2013, GZU; 200 μm); **j**, *Trapelia glebulosa* (Montana, S/09.2013, photo courtesy of Tim Wheeler: ca. 1 mm); **k**, *Placopsis cribellans* (*upper right*) and *P. lambii* (*bottom left*; Alaska, S/09.2014, GZU: 1 mm); **l**, **Baeomycetaceae**. L, *Parainoa subconcolor* (Italy, Arnold Lich. Exs. 938, GZU: 500 μm), the only trapelioid species recovered outside of Xylographaceae and Trapeliaceae

Early concepts of the phylogenetic relationships of lichenized fungi drew heavily on the shape and gross attributes of ascomata, ascospores and thallus and the photobionts with which they associate (Watson 1929). Starting in the 1960s, detailed anatomical studies of the ascus (Letrouit-Galinou 1966; Hafellner 1984), ascomatal ontogeny (Letrouit-Galinou 1968) and secondary metabolite chemistry (Culberson 1969), as well as increased openness to the possibility of convergent evolution, led to a shake-up in the classification of lichenized fungi. One of the numerous enduring legacies of this era is the recognition that emerged in the 1970s and 1980s of the close relatedness of a group of genera with a characteristic non-amyloid, unitunicate ascus and well-defined apical cushion that came to be called the *Agyrium*- or *Trapelia*-type ascus (Hertel 1970). Using mainly ascus and ontogenetic characters, Lumbsch (1997) proposed uniting 16 of these genera into Lecanorales suborder Agyriinae, which was subsequently raised to the level of its own order, Agyriales (Lumbsch et al. 2001a). However, with the application of molecular phylogenetics to more members of this group it became apparent that ascus characters and ontogeny also exhibit convergent evolution, and that several of these genera are only distantly related, including *Anzina* and *Elixia* (Wedin et al. 2005), *Miltidea* (Widhelm and Lumbsch 2011) and not least the name-giving genus *Agyrium* (Lumbsch et al. 2007a). This latter finding resulted in the taxonomic orphaning of the genera remaining and led to several new taxonomic proposals, partly reflecting renewed attention to relationships with Baeomycetaceae (e.g., Lumbsch et al. 2007a; Lumbsch and Huhndorf 2010; Hodkinson and Lendemer 2011). The rump group can now be considered to consist of 11 genera (Trapeliaceae sensu Lumbsch and Huhndorf 2010): *Amylora*, *Coppinsia*, *Lambiella* (Spribille et al. 2014), *Lithographa*, *Placopsis* (encompassing *Aspiciliopsis* and *Orceolina*), *Placynthiella*, *Ptychographa*, *Rimularia*, *Trapelia*, *Trapeliopsis* and *Xylographa* (*Sarea* was recently excluded by Miadłikowska et al. 2014). Several of these genera were included in Lecanoromycetes subclass Ostropomycetidae at the time it was first recognized (Miadłikowska and Lutzoni 2004) and have since been routinely included in phylogenetic hypotheses of that subclass. For the purposes of the present discussion we will refer to this group as the trapelioid fungi (Fig. 1).

Several characteristics suggest that trapelioid fungi are a promising study system for evolutionary biology of the lichen symbiosis, namely their role as pioneer colonizers (e.g., Jahns 1982; Ullmann et al. 2007; Raggio et al. 2012), their photobiont diversity (Voytsekhovich et al. 2011), and their substrate specificity (Spribille et al. 2008, 2014). Developing them as a model system however requires resolving evolutionary relationships that until now have been deeply entangled with other clades of Ostropomycetidae. Since its recognition as a subclass by Reeb et al. (2004), nearly all phylogenies of Ostropomycetidae have recovered a pattern of resolved terminal clades and an unresolved backbone (Lumbsch et al. 2005: mtSSU, nuLSU; Schmitt et al. 2005: mtSSU, nuLSU; Wedin et al. 2005: mtSSU, nuLSU; Lumbsch et al. 2007c: mtSSU, nuLSU; Schmitt et al. 2010: nuLSU, mtSSU, RPB1, MCM7; Lumbsch et al. 2012: mtSSU, nuLSU; Bendiksby and Timdal 2013: ITS, mtSSU, nuLSU; Otálora and Wedin 2013: mtSSU, RPB1, MCM7; Prieto and Wedin 2013: nuSSU, nuLSU, 5.8S, mtSSU, RPB1, MCM7 and Prieto et al. 2013, same loci). The pattern of persistent low backbone support is perhaps best visualized in the large-scale phylogeny of the group presented by Miadłikowska et al. (2014, Fig. 2). These results convinced us that any resolution of deep relationships of trapelioid fungi would require a taxon sampling that encompassed representatives of all key clades and more loci than anything sampled to date.

**Fig. 2:**
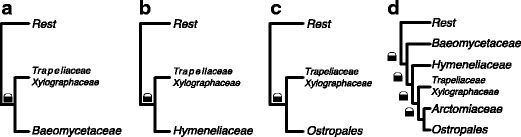
Phylogenetic hypotheses tested using the SOWH test. Locks represent constrained nodes. A: Hypothesis as in Lumbsch et al. (2007a) with Baeomycetaceae being sister to trapelioids, B: Hypothesis of a sister group relationship of trapelioids and Hymeneliaceae, C: Hypothesis of sister group relationship of trapelioids and Ostropales. D: Hypothesis obtained by Miadłikowska et al. (2014) with Ostropales and Arctomiaceae forming the crown group in Ostropomycetidae

Aside from trapelioid fungi, the Ostropomycetidae are dominated by two species-rich main groups, usually treated as orders, namely the Ostropales, which have almost always been recovered as monophyletic (Kauff and Lutzoni 2002; Miadłikowska et al. 2006, 2014; Lumbsch et al. 2007b; Prieto and Wedin 2013; Prieto et al. 2013), and the Pertusariales, which usually have not (e.g., Wedin et al. 2005; Lumbsch et al. 2007a, 2007b; Prieto et al. 2013; Prieto and Wedin 2013; but see Miadłikowska et al. 2014). Five smaller “floating clades” also feature in most studies: Arctomiaceae, Baeomycetaceae, Hymeneliaceae, Sarrameanaceae and Schaereriaceae. Phylogenetic hypotheses including trapelioid fungi repeatedly recover three recurring motifs that, though mostly lacking statistical support and in some cases forming polytomies, form the working basis for evolutionary hypotheses in this group:

1. trapelioids are sister to the Baeomycetaceae, with or without the Hymeneliaceae (Wedin et al. 2005, as Agyriales; Lumbsch et al. 2007a, as Agyriaceae core group, and Lumbsch et al. 2007b, as Agyriales; Lumbsch et al. 2007c; Bendiksby and Timdal 2013);
2. trapelioids are sister to the Ostropales or sandwiched between the Ostropales and *Baeomyces* + *Arctomia* (Miadłikowska et al. 2006; Prieto et al. 2013; Prieto and Wedin 2013);
3. trapelioids are sister to the Ostropales + Arctomiaceae (Miadłikowska et al. 2014, as Trapeliales); this is the only study to present statistical support for multiple relationships.

The lack of support until now for sister group level relationships in the vicinity of trapelioid fungi makes it impossible to confidently infer order of divergence, and by extension character evolution, in this speciose group. A deeper locus sampling is in our view the only way a confident assessment of evolutionary relationships of trapelioid fungi can be advanced. We accordingly set out to assemble a large data set of trapelioid fungi as well as obtain an eight-locus sampling for each of the “floating clades” in Ostropomycetidae and selected outgroups. In so doing, we added hundreds of newly generated sequences and carefully curated published sequences to weed out mixed accessions (sequences of one species derived from different vouchers) that have weakened previous phylogenetic analyses. Our goals were: 1) to resolve as far as possible the backbone of the Ostropomycetidae; 2) test the support for rejecting alternative hypotheses about relationships that have been proposed to date for Trapeliaceae, Baeomycetaceae, Hymeneliaceae and Arctomiaceae; and 3) adjust the taxonomy to reflect some of the statistically significant evolutionary inferences derived from our analyses.

## Material and methods

### Assembly of taxon sample set

We designed our taxon sample to include representatives of all described orders of Ostropomycetidae and all available genera of trapelioid lichenized fungi. We drew upon two sources of DNA sequences. First we screened Genbank for specimens from which multiple loci had been sequenced; we did not permit mixed accessions (sequences attributed to one species but derived from different specimens) because the often dynamic understanding of species delimitations can lead to seemingly congruent sequences, if acquired from different isolates, actually deriving from different species. Second, because no Genbank samples had all eight loci targeted for this study, we ended up extracting DNA from fresh material from every taxonomic order of Ostropomycetidae. Consistent with our focus on trapelioid fungi we accorded most attention to the 11 genera of trapelioid lichenized fungi until now assigned to Trapeliaceae by Lumbsch and Huhndorf (2010; see Introduction). We also invested considerable sequencing effort in other groups within Ostropomycetidae. We did not undertake resampling of speciose families such as Graphidaceae, Megasporaceae and Pertusariaceae s.lat. that have been found to be monophyletic in the past (Mangold et al. 2008; Rivas Plata et al. 2013; Nordin et al. 2010; Schmitt and Lumbsch 2004; Schmitt et al. 2006, 2010). Instead, we tried to obtain as many loci as possible for several members of every major group, including where necessary from new isolates. Similarly, we generated multilocus data sets for single to multiple species in Lecanoromycetidae and Umbilicariomycetidae for use as outgroups. Newly generated sequences and used Genbank accessions are summarized in Table 1. Detailed information on isolated DNA vouchers, their obtained loci and NCBI accession numbers is provided in Online Resource 1.

**Table 1:**
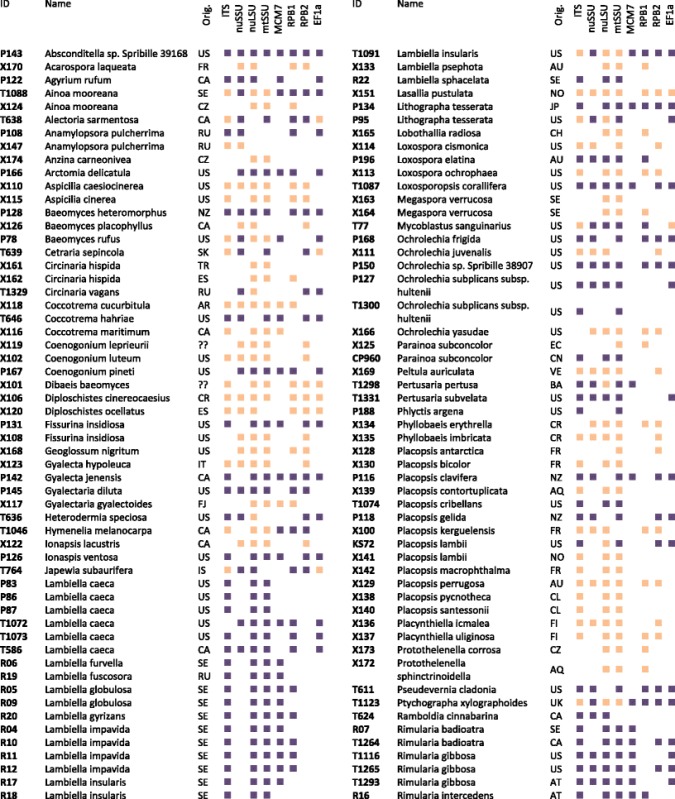

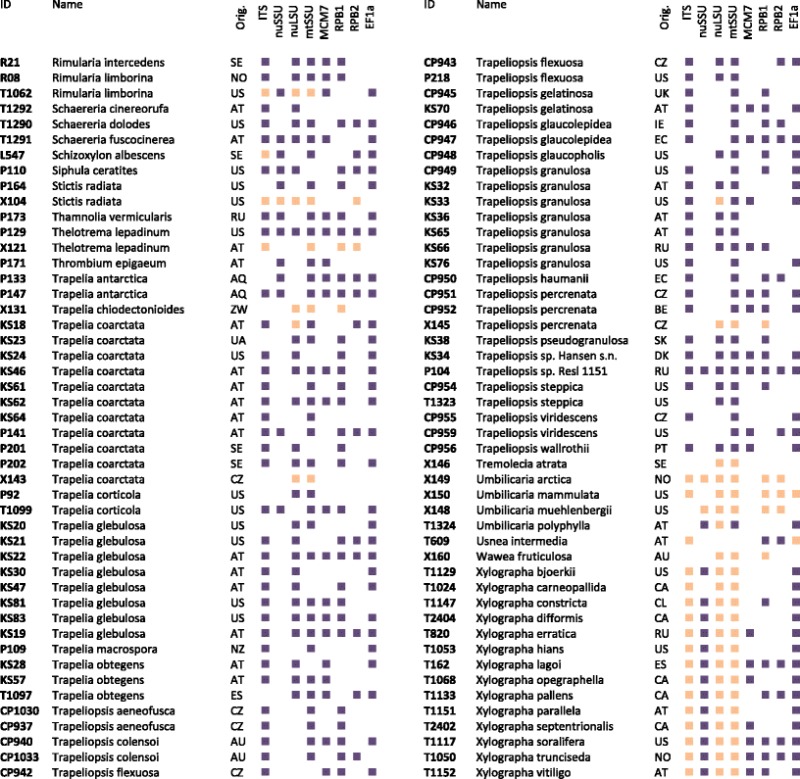
Species, origin and loci of vouchers used in our study. ID indicates lab tracking numbers that cross-reference with Fig. 4 and Online Resource 1. Purple squares indicate newly published sequences, cream-colored squares (and all IDs beginning with “X”) refer to previously published Genbank accessions. Country codes under “Orig.” follow internationally standardized two-letter abbreviations. For more detail on the used specimens, including geographical provenance and NCBI accession numbers, refer to Online Resource 1

### DNA acquisition, polymerase chain reaction and Sanger sequencing

Samples from ascomata or thallus fragments were pulverized in a Retsch cell grinder in 1.5 ml Eppendorf tubes with a single 3 mm steel bead after freezing at −80 °C. Lysis buffer was applied directly to the sheared cells. Further extraction of genomic DNA was performed using the Qiagen DNeasy Plant Mini kit following the manufacturer’s instructions. For sparse specimen material the QIAmp DNA Investigator Kit was used. We eluted raw nucleic acids in 50 to 75 μL of elution buffer without RNAse treatment. Undiluted samples were used for downstream PCR reactions. For each sample, we sequenced as many as possible of eight commonly used gene fragments: the internal transcribed spacer regions 1 and 2 as well as the embedded 5.8S region of the ribosomal rDNA (hereafter ITS); the nuclear ribosomal large subunit (nuLSU); the nuclear ribosomal small subunit (nuSSU); the mitochondrial small ribosomal subunit DNA (mtSSU); parts of the largest and second largest subunit of the RNA polymerase II (RPB1 and RPB2, respectively); part of DNA replication licensing factor minichromosome maintenance complex 7 (MCM7); as well as a commonly used partial sequence of transcription elongation factor 1 alpha (EF1a). Primers and annealing temperatures used are listed in Table 2. PCR was performed using PuReTaq Ready-To-Go PCR beads. After checking the size of the obtained fragments on ethidium bromide-stained agarose gels, we purified PCR products using the AMPure XP bead clean-up protocol, or the Omega E.Z.N.A. Cycle Pure Kit according to manufacturer’s instructions. Purified PCR products were than sequenced by Microsynth (Switzerland).

**Table 2:** PCR primers used in this study

Name	Sequence (5′–3′)	Annealing temp (°C)	Citation
ITS1F	CTTGGTCATTTAGAGGAAGTAA	52	Gardes and Bruns 1993
ITS4	TCCTCCGCTTATTGATATGC	52	White et al. 1990
NS20 (nuSSU-0072)	CATGTCTAAGTTTAAGCAA	53	Gargas and Taylor 1992
NS1	GTAGTCATATGCTTGTCTC	53	White et al. 1990
NS17 (nuSSU-0852)	CGTCCCTATTAATCATTACG	53	Gargas and Taylor 1992
LR0R	ACCCGCTGAACTTAAGC	52	Vilgalys unpublished
LR7	TACTACCACCAAGATCT	52	Vilgalys and Hester 1990
LR4_Trap	TTTGCACGTCAGAACCGCTGCG	52	Spribille et al. 2014
LRascF	CCTCAGTAACGGCGAG	56	Schneider et al. 2015
LRascR	AGGCTTCGTCACGGAC	56	Schneider et al. 2015
mrSSU1	AGCAGTGAGGAATATTGGTC	52	Zoller et al. 1999
mrSSU3R	ATGTGGCACGTCTATAGCCC	52	Zoller et al. 1999
RPB1-VHAFasc	ADTGYCCYGGYCATTTYGGT	52	Hofstetter et al. 2007
RPB1-Cr	CCNGCDATNTCRTTRTCCATRTA	52	Matheny et al. 2002
fRPB2-5 F	GAYGAYMGWGATCAYTTYGG	52	Liu et al. 1999
fRPB2-7CR	CCCATRGCTTGYTTRCCCAT	52	Liu et al. 1999
MCM7-709for	ACIMGIGTITCVGAYGTHAARCC	50	Schmitt et al. 2009
MCM7-1348rev	GAYTTDGCIACICCIGGRTCWCCCAT	50	Schmitt et al. 2009
EF-983f	GCYCCYGGHCAYCGTGAYTTYAT	56	Rehner and Buckley 2005
EF-1567R	ACHGTRCCRATACCACCRATCTT	56	Rehner and Buckley 2005
Efdf	AAGGAYGGNCARACYCGNGARCAYGC	56	Rehner unpublished
EF-1953-R	CCRGCRACRGTRTGTCTCAT	56	Rehner unpublished

### Alignment

Sequence alignment was performed using MAFFT v7 (Katoh and Standley 2013). MAFFT allows the use of different alignment algorithms depending on the properties of input sequences (e.g., presence of unalignable introns). We used the –genafpair flag to align ribosomal ITS, nuSSU, nuLSU and mtSSU sequences and the –globalpair algorithm to align protein-coding MCM7, RPB1, RPB2 and EF1a sequences. We set MAFFT to run 10,000 iterations for each alignment. Alignments were manually checked for obvious errors and corrected when needed. Embedded sequence alignment and subsequent concatenation were performed in a single pipeline using custom Python scripts.

With a custom Python script we eliminated intron positions on the basis of the relative presence of nucleotides at each position in the alignment. We applied a cut-off value of 10 %, so that positions with more than 90 % missing data were excluded. This alignment was used for all subsequently performed phylogenetic analyses. To provide information on the completeness of our alignment we created a visualized alignment plot in which the percent completeness of each individual nucleotide position is graphically displayed relative to the number of isolates included in the alignment. The nucleotide completeness matrix was retrieved with a custom Python script and depicted using scripts written in the graphic programming language Processing 2. All used Python scripts have been released on the GitHub page of the first author under the repository phylo-scripts v0.1 (Resl 2015, https://github.com/reslp).

### Phylogenetic analyses

We performed maximum likelihood (hereafter ML) as well as Bayesian inference (hereafter BI). The ML phylogenetic analysis was performed using RAxML v8.0.4 (Stamatakis 2014) and BI was carried out using MrBayes 3.2.2 (Ronquist and Huelsenbeck 2003). We created partitions for each gene fragment as well as for an intron present in RPB1 in the original alignment. Protein-coding genes were partitioned according to codon position. This a-priori selected scheme was used as input for PartitionFinder 1.1.1 (Lanfear et al. 2012) to optimize partitions and substitution models. As input parameters we selected linked branch lengths and the Bayesian Information Criterion (BIC) as optimality criterion in a greedy search. PartitionFinder retained ten partitions and chose GTRGAMMAI for each. To evaluate statistical node support we generated 1000 bootstrap replicates of the alignment using the fast bootstrap option of RAxML. We performed a maximum likelihood search to find the best scoring tree according to its log likelihood score (RAxML option –f a). To check for topological conflicts, we created single locus trees using RAxML. For each gene we used the GTRGAMMAI substitution model and generated 500 bootstrap replicates. The maximum likelihood search was performed in the same way as for the concatenated dataset. Topological conflict in single gene trees was assessed with the software compat.py (Kauff and Lutzoni 2003) for a cut-off bootstrap value of 70.

We performed BI to provide a second topological hypothesis of the concatenated dataset. As in the ML analysis we used the partitioning scheme selected by PartitionFinder. Parameters of the DNA substitution model for each partition were estimated by MrBayes using reversible jump MCMCMC as implemented in the command lset nst = 6. Bayesian phylogenetic analyses are known to have problems reaching stationarity when analyzing large datasets owing to the limitations of low run and chain numbers to adequately explore potential parameter space (Hackett et al. 2008). Preliminary Bayesian analyses of our dataset with two independent runs and four chains each failed to converge even after 100 million generations (mean deviation of split frequencies remained > 0.05) with the standard temperature factor of the heated chain set to 0.2. We substantially improved the diagnostic metrics (standard deviation of split frequencies, ESS values of parameter estimates of the model) of our analyses by performing four independent MCMCMC runs with eight chains each for 80 million generations. To provide better chain mixing we further set the temperature factor of the heated chain to 0.3 and increased the number of swaps to two. We used a 30 % relative burn-in (relburnin = yes burninfrac = 0.30) and the flag contype = halfcompat in sumt to create a majority rule consensus tree. We checked for convergence of the MCMCMC runs in terms of the obtained topology using the web version of AWTY (Wilgenbusch et al. 2004). We also investigated the convergence of the parameter estimates of the runs by Tracer 1.6 (Rambaut et al. 2014). In both cases we used a burn-in proportion of 30 %. The final tree was visualized in R using the ape package (R Development Core Team 2013).

### Testing topological hypotheses

We performed topology tests on four alternative phylogenetic hypotheses specific to the immediate sister group relationships of trapelioid fungi (Fig. 2): A) the backbone topology of trapelioid fungi and Baeomycetaceae recovered by Lumbsch et al. (2007a), the 1:1 sister group relationship of trapelioid fungi to B) Hymeneliaceae and C) Ostropales, and finally D) the backbone obtained for trapelioid fungi and neighbouring groups by Miadłikowska et al. (2014). Two of these hypotheses (A, D) were formulated a priori but the others (B, C), as well as later specific alternative hypotheses constraining monophyly of two genera (see Discussion), were developed in part *a posteriori* after studying our own and past phylogenies. All tested scenarios focus on nodes that lack support in both phylogenetic reconstructions, BI and ML respectively. Bayesian phylogenetic methods are known to overestimate support from concatenated alignments relative to bootstrap methods applied in maximum likelihood analysis, which tend to be more conservative (e.g., Suzuki et al. 2002). Consequently, nodes that were supported in BI but not in ML were of particular interest to us. In keeping with a more conservative likelihood approach for hypothesis testing we applied the Swofford-Olsen-Waddell-Hillis (hereafter SOWH) test to each scenario. The SOWH test allows a direct comparison between an *a posteriori* obtained topology and a priori developed phylogenetic hypotheses (Swofford et al. 1996). It is thus more appropriate than the Approximately Unbiased (AU)- or Kishino-Hasegawa (KH)-tests, which assume strict a priori hypotheses (Goldman et al. 2000).

The SOWH tests were implemented in the SOWHAT pipeline (Church et al. 2014, https://github.com/josephryan/SOWHAT). The pipeline relies on RAxML for generating phylogenetic analyses and SeqGen (Rambaut and Grassly 1997) for creating simulated alignments. Statistical tests are performed using R. The test involves generating a null distribution of the differences in likelihood of the constrained and unconstrained topology by parametric sampling of simulated alignments that fit the original topology parameters (branch lengths, substitution model). The difference in log-likelihood of the constrained and unconstrained tree of the original alignment (test-statistic) is compared to the obtained null distribution of log-likelihood differences from simulated alignments with a one-sided *t*-test. The obtained p-value of the test indicates the probability that the observed difference in likelihood values would also be observed under H_0_ (no difference between both topologies). For each test we used RAxML in the PTHREADS version and employed a GTRGAMMA substitution model for all partitions as described above. Different numbers of trees were calculated depending on the minimum number needed for completing a null distribution relative to the data. We used the built-in convergence assessment algorithm (flag –stop) to halt the analysis when it reaches a point where subsequent sampling is unlikely to alter the likelihood distribution (Church et al. 2014). Statistical support to reject the alternative hypothesis was considered sufficient if *p* < 0.05. The specific Newick coding of topological constraints is provided in Online Resource 2.

## Results

### Acquired sequences

**Fig. 3:**
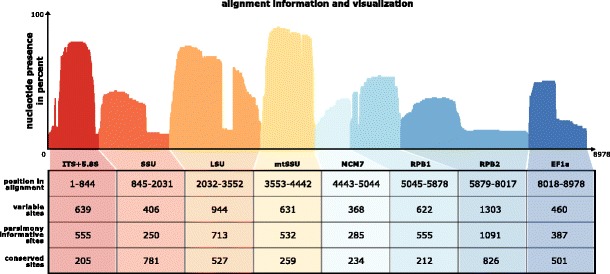
Visualized alignment plot indicating percent completeness of nucleotides per alignment position (*above*) and the number of variable, parsimony-informative and conserved sites per gene fragment (*below*). x-axis: nucleotide position in alignment. y-axis: percent nucleotide completeness

We obtained a total of 657 new sequences from 148 isolates including all nine trapelioid genera from which we had fresh material; only *Amylora* and *Coppinsia* could not be sampled. We acquired the most sequences for mtSSU and the fewest for RPB2 (Online Resource 1). We obtained five or more loci for 64 isolates. Together with 309 sequences from Genbank, we incorporated 966 sequences from 205 isolates into our alignment (Table 1; Online Resource 1). The raw alignment consisted of 20,999 positions. Following removal of sites with missing data exceeding threshold values, the final alignment used for phylogenetic analyses consisted of 8978 positions including introns in the ITS and nuLSU and nuSSU region as well as in the RPB1 gene (Fig. 3). The original alignment has been deposited at TreeBASE under study ID 16680.

### Phylogenetic hypotheses

The best-scoring ML tree had a Ln of −184,362.3943. For BI we allowed the run to continue open-ended until the average deviation of split frequencies had stabilized under 0.016, which happened after 14 million generations. We then let the analysis run for another two million generations to ensure that the chains were not trapped in sub-optima of topology space. The final average standard deviation of split frequencies was 0.013827. The AWTY plots (Online Resource 3) show the posterior probabilities of splits over all pairs of independent MCMC runs indicating convergence of the topology. Tracer showed convergence of the LnL values of the tree (Online Resource 4) as well as for all parameters in the explored parameter space (effective sample size > 200; data not shown).

Tests for topological incongruence showed several disagreements between gene trees (Online Resource 5a–h). After manually investigating each conflict reported by compat.py most were found to be due to sequence gaps in either of the two trees or related to shallow relationships (e.g., affect species-level relationships). The remaining conflicts we found are summarized in Online Resource 6. Since removing those sequences did not affect nodal resolution, we retained them in the dataset.

**Fig. 4:**
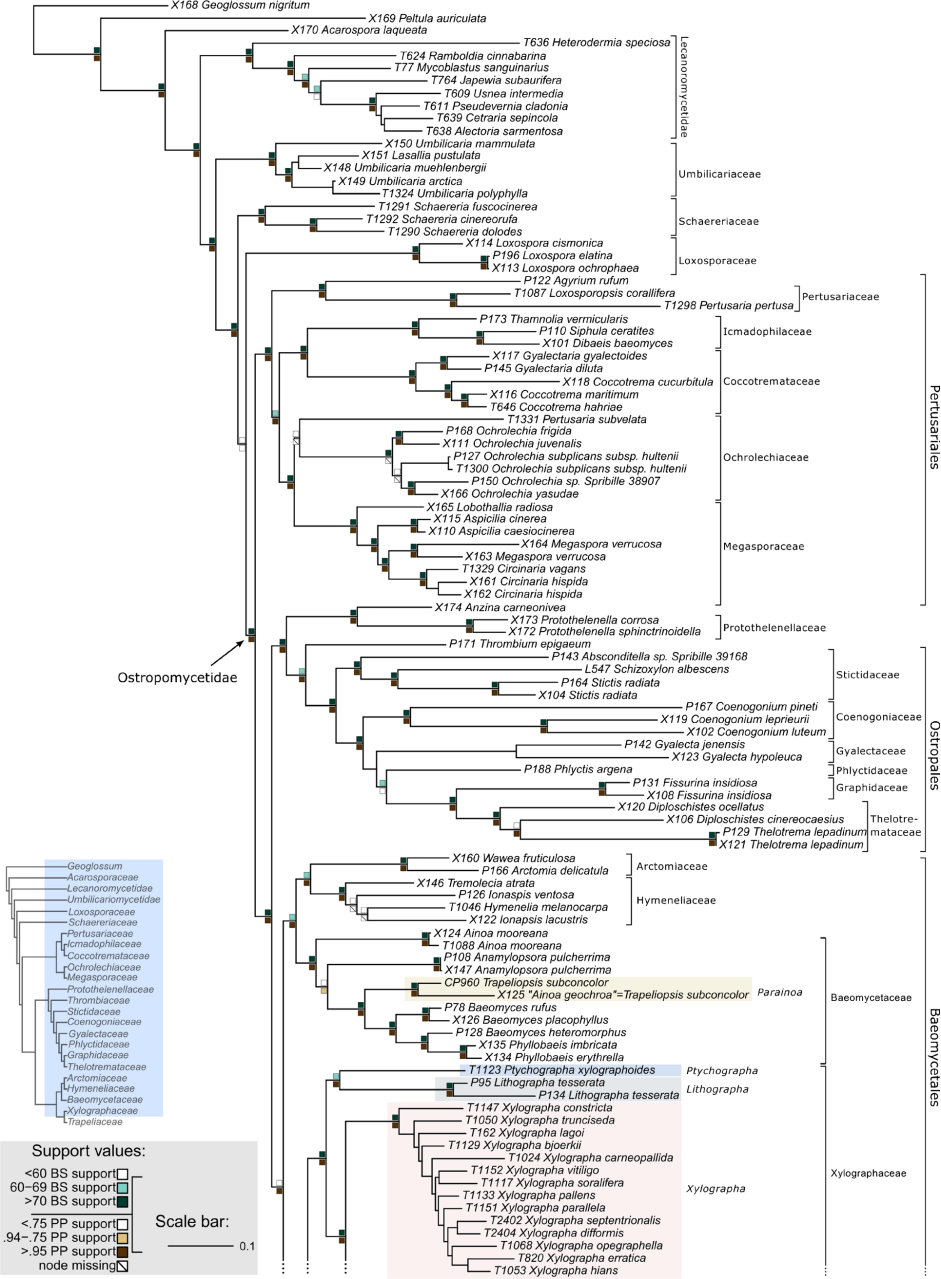

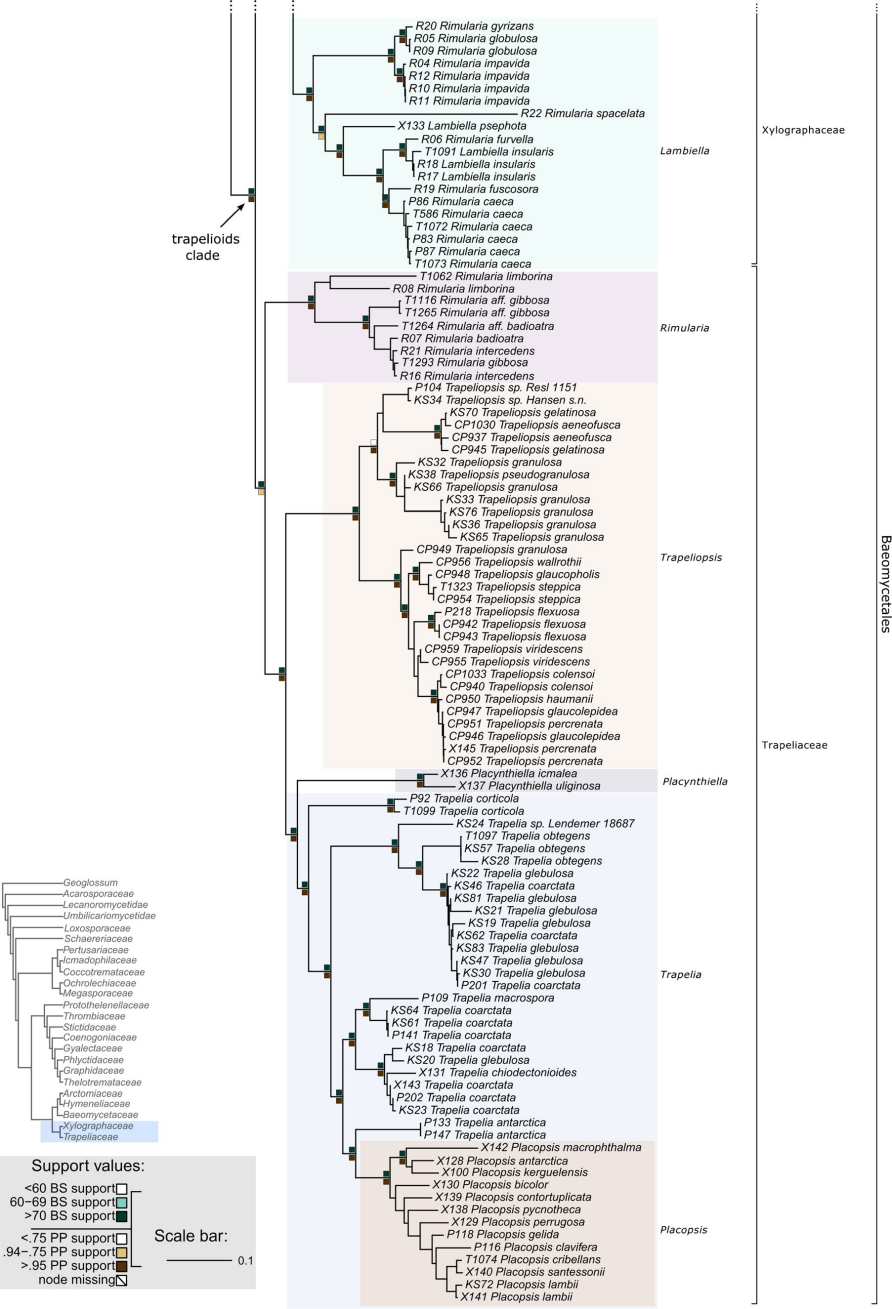
Estimated tree of Ostropomycetidae obtained from concatenated maximum likelihood analysis of eight fungal gene fragments. Boostrap support values are plotted as boxes above, Bayesian posterior probabilities as boxes below nodes. Trapelioid groups are demarcated with colored boxes

ML and BI analyses of the concatenated dataset yielded similar topologies and we plot node support of both ML and BI analyses on the best-scoring ML topology (Fig. 4; all values in Online Resources 7–9). The Ostropomycetidae form a well-supported, reciprocally monophyletic clade with the outgroups (87%BS / 1.00PP support) if circumscribed to exclude Loxosporaceae and Schaereriaceae. All nine trapelioid genera from which we obtained sequences resolved as a monophyletic clade (100%BS / 1.00PP support; Fig. 4b), which in turn splits into two deeply divergent, reciprocally monophyletic clades. The only trapelioid found outside of this group was *Trapeliopsis subconcolor*, which was recovered within a strongly supported (100%BS / 1.00PP) aeomycetaceae. The latter forms a clade only supported in BI (60%BS / 1.00PP; together the “BAH clade”) including Arctomiaceae and Hymeneliaceae, each of which are independently strongly supported in both analyses (Fig. 4a). The BAH clade forms a supported sister group to trapelioids only in BI (45%BS / 0.95PP) Ostropales are resolved in a monophyletic clade (91%BS / 1.00PP support) that forms an unresolved sister group relationship with *Protothelenellaceae*. The split between the trapelioid/BAH clade and the Ostropales/*Protothelenellaceae* clade is supported in both analyses (73%BS / 0.98PP). The Pertusariales clade forms a monophyletic group (73%BS / 1.00PP) that is reciprocally monophyletic to the rest of Ostropomycetidae. The original Bayesian topology as well as all ML gene trees are provided in Online Resources 5a–h and 7–9.

Within trapelioids, the genus *Rimularia* s.lat was found to be polyphyletic, with most sampled species coming out in a monophyletic clade with *Lambiella* (97%BS / 1.00PP), while the type species, *R. limborina*, is recovered in a monophyletic clade sister to a well-supported clade including *Placopsis*, *Trapelia*, *Trapeliopsis* and *Placynthiella* with 100%BS / 1.00PP (Fig. 4b). *Trapelia* consists of four well-supported clades that form a paraphyletic assemblage with *T. corticola* as its most basal member (89%BS / 0.99PP). *Placopsis* is recovered as monophyletic but nested within *Trapelia*. The recently described *Trapelia antarctica* forms a well-supported sister group relationship with *Placopsis. Trapeliopsis* is monophyletic as currently circumscribed (100%BS / 1.00PP) with the exception of *T. subconcolor*, which comes out with high support in Baeomycetaceae and forms a well-supported clade with the Genbank-derived isolate X125 “*Ainoa geochroa*”, which is in fact also *T. subconcolor* (see Discussion). Within the Baeomycetaceae, *Baeomyces* is paraphyletic with *Phyllobaeis*.

### Topology tests

Table 3 summarizes the results obtained by the individual SOWH test scenarios for four different topological scenarios that account for previously formulated phylogenetic hypotheses of sister group relationships of trapelioid clades (Fig. 2). Scenarios A and B tested for sister group relationships of trapelioid groups (trapelioids/Baeomycetaceae/Hymeneliaceae). Under the test for scenario A, a sister group relationship of trapelioids and Baeomycetaceae could not be rejected (*p* = 1). Under scenario B, a sister group relationship between trapelioids and Hymeneliaceae the SOWH test was not able to provide a significant solution given our data. Scenario C tested a sister group relationship of trapelioids and Ostropales, which is strongly rejected (*p* < 0.01). Scenario D, in which Ostropales/Arctomiaceae are sister to trapelioids, is rejected with high confidence given our dataset (*p* = 0).

**Table 3:** Results of SOWH topology tests

Scenario	ML value of best tree	ML value of best tree w/ constraints	Test statistic	Size of null distribution	Percent ratio	Parametric *p*-value
Scenario A	−184,260.479773	−184,254.136599	−6.343174	158	50	1
Scenario B	−184,260.479773	−184,264.800805	4.321032	852*	0.821*	0.7162547*
Scenario C	−184,260.479773	−184,292.915605	32.43583	208	50	2.833279e-152
Scenario D	−184,260.479773	−184,301.675029	41.19526	212	50	0
Ainoa - Parainoa	−184,260.479773	−184,896.651532	636.1718	194	50	0
Rimularia - Lambiella	−184,260.479773	−184,357.783154	97.30338	152	50	0

Scenarios A–D refer to hypotheses in Fig. 2. Ainoa-Parainoa and Rimularia-Lambiella refer to tests for monophyly of genera that are shown to be polyphyletic in our concatenated analysis. ML value of best tree: LnL value of the unconstrained tree obtained by maximum-likelihood analysis. ML value of best tree w/constraints: LnL value obtained by maximum-likelihood analysis for the constrained scenario. Test statistic: LnL difference between both trees. Size of null distribution: Number of tree pairs for which likelihood differences were obtained. Percent ratio: Indicates sampling completeness of the null-distribution of the test

Parametric *p*-value: Probability for H_0_ (no difference between topologies)

* Value when test was terminated

## Discussion

### The sister group relationships of trapelioid fungi

Our phylogenetic hypothesis for Ostropomycetidae is the first to recover support for nearly the entire backbone of the subclass in both ML and BI. Amongst other things, it solidifies an emerging pattern, first observed by Miadłikowska et al. (2014) in a five-locus sample, in which a monophyletic Pertusariales is sister to the rest of the subclass. It confirms a monophyletic Ostropales as in previous studies, and is the first to recover a well-supported monophyletic group for a comprehensive sampling of trapelioid genera. In the process, it tightens a circle around three nodes that remain problematic inasmuch as they are lacking support in ML or BI and thus constitute the remaining destabilizing elements in the phylogeny: 1) the relationship between Baeomycetaceae and Arctomiaceae/Hymeneliaceae (the “BAH clade”); 2) the relationship between the latter two families (Arctomiaceae and Hymeneliaceae) individually; and 3) the immediate sister group relationship of trapelioids to the BAH clade. These three groups interact with each other and almost certainly account for most of the discrepancies in node support in our analyses.

Even without full resolution in our phylogenetic hypothesis, not all of the sister group relationships that have been hypothesized in previous studies are equally probable. The hypothesis of a sister group relationship to Ostropales can be rejected with *p* < 0.01 given the taxon and locus sampling used here. Similarly, a topology consistent with the hypothesis in which trapelioids are sister to a combined Ostropales + Arctomiaceae and these in turn sister to Hymeneliaceae and Baeomycetaceae, is impossible to obtain with our data set and can be ruled out (*p* = 0). This leaves the first and original set of hypotheses based on molecular data, namely a sister group relationship to Baeomycetaceae and/or Hymeneliaceae, with or without Arctomiaceae. Constraining trapelioids to form a single monophyletic sister group relationship with either Baeomycetaceae or Hymeneliaceae yields a likelihood distribution not significantly different from the unconstrained topology, meaning that the null hypothesis cannot be rejected (Table 3; we did not test the 1:1 sister group relationship to Arctomiaceae because of the small sample).

### Major groups of trapelioid fungi

The nine genera of trapelioids resolve into two reciprocally monophyletic clades representing *Lithographa*, *Ptychographa*, *Xylographa* and *Lambiella*, on the one hand, and *Rimularia*, *Placynthiella*, *Trapeliopsis*, *Trapelia*, and *Placopsis*, on the other. The first grouping includes mostly species with linearized, hysteriothecial ascomata (though round ascomata also occur, e.g., in *Lambiella caeca*: Fig. 1f) and we recognize this group as the family Xylographaceae (see below). The other represents mainly species with rounded ascomata, the Trapeliaceae in the original sense of Hertel (1970). The split between Xylographaceae and Trapeliaceae runs through the middle of the genus *Rimularia* as used by current authors, as well as through the family Rimulariaceae (Hafellner 1984; Hertel and Rambold 1990). The split echoes earlier suspicions by Hertel (1984) that *Rimularia* consists of disparate elements. *Rimularia* s.str. (around the type species *R. limborina*) forms a basal group with Trapeliaceae, though supported only in the Bayesian analysis. The majority of species sampled thus far go to Xylographaceae, where the name *Lambiella* was established for *L. psephota* by Hertel (1984). The polyphyly of *Rimularia* as defined to date is well supported and statistically beyond doubt (result of parametric topology test: *p* = 0). Notably, *Lambiella* in its expanded definition adopted here itself splits into deeply diverging lineages, with a clade for the *impavida* group, a clade for the *insularis* group and two isolated branches supporting the type species of *Lambiella*, *L. psephota*, as well as *L. sphacelata*; overall, however, it is monophyletic.

Within the Trapeliaceae, the genus *Trapelia* is strongly paraphyletic with *Placopsis* nested within *Trapelia* as currently understood. The paraphyly also extends to the species level, with taxa such as *T. coarctata* and *T. glebulosa* recovered in disparate clades. The genus is the subject on ongoing character evolution studies (K. Schneider, in prep.).

### Novelties in non-trapelioid Ostropomycetidae

A collateral consequence of expanding taxon and locus sampling is the resolution of several relationships that have long been unstable in Ostropomycetidae, beyond the sister group relationships of the trapelioid genera. The position of Schaereriaceae, represented by *S. corticola* and/or *S. fuscocinerea*, has typically been plotted as the first divergence in Ostropomycetidae, though always without support (Wedin et al. 2005; Miadłikowska et al. 2006; Lumbsch et al. 2007a, b). Ours is the first phylogeny to include the type species, *Schaereria cinereorufa* (Hafellner 1984). Miadłikowska et al. (2014) included *Schaereria* together with *Loxospora* in Sarrameanaceae, though they conceded that the anatomical evidence made this seem unlikely. We also provide sequences of the type species of *Loxospora*, *L. elatina*, for the first time, confirming its close relationship to other species placed in that genus, and retain it in Loxosporaceae in absence of evidence supporting its relationship to *Sarrameana*. Though statistically testing this was outside the scope of this study, both of our phylogenetic hypotheses suggest a close relationship of *Schaereria* to *Loxospora* could be rejected, as well as their inclusion in an otherwise monophyletic Ostropomycetidae. The Pezizalean-like ascus of *Schaereria* (Hafellner 1984; Lumbsch 1997) has few if any parallels in this subclass. Similarly, the unstable position of *Loxospora* even in a six-locus sample suggests an isolated position.

The recovery of *Anzina* as sister to *Protothelenella* at the base of Ostropales sheds light on another heretofore unstable element in the phylogeny of Ostropomycetidae. *Anzina* was repeatedly postulated to belong to Trapeliaceae based on similarities in ascus structure, conidiogenesis and secondary metabolites (Scheidegger 1985; Lumbsch 1997). Both *Anzina* and *Protothelenella* were sequenced and recovered as sister to Ostropales in separate studies in 2005 (*Anzina*: Wedin et al. 2005; *Protothelenella*: Schmitt et al. 2005). *Protothelenella* was subsequently recovered on a polytomy together with Arctomiaceae, Schaereriaceae and Ostropales by Lumbsch et al. (2007a) and even on a polytomy with what are here called trapelioid fungi by Lumbsch et al. (2007b, 2007c). Curiously neither genus was included in a later overview of secondary delichenization in Ostropales (Baloch et al. 2010). Lumbsch et al. (2012) recovered both on a supported branch in a two locus (mtSSU, nuLSU) phylogeny of Ostropomycetidae. Our results appear to lend strong support to the monophyly of *Anzina* and *Protothelenella* and further suggest that the synonymization of Thrombiaceae and Protothelenellaceae by Schmitt et al. (2005) should be revisited in future sampling at the base of the Ostropales.

### Insights into lower level relationships in trapelioid fungi

1. We found no support for rejecting the placement of *Anamylopsora* in Baeomycetaceae. *Anamylopsora* was originally assigned to Baeomycetales on the basis of morphological characters, but in its own family, Anamylopsoraceae. In describing the family, Lumbsch et al. (1995) pointed out similarities in ascomatal ontogeny, excipular structures and the form of conidia of *A. pulcherrima* with Baeomycetaceae. However, differences in the ascus apical apparatus, the structure of the cortex and the production of benzyl esters led Lumbsch et al. (1995) to the conclusion that similarities between *Anamylopsora* and Baeomycetaceae must be due to convergent evolution. Because all heretofore published sequences of *A. pulcherrima* were derived from a single individual (Lumbsch et al. 2001b, 2005), we generated sequences from four loci from a second specimen. Orthologous DNA sequences closely match those obtained by Lumbsch et al. (2001b) and group in a monophyletic Baeomycetaceae.
2. We recover *Ainoa* in Baeomycetaceae and found one set of previous Genbank accessions to contain errors. The genus *Ainoa* was erected by Lumbsch et al. (2001b) with the type species *Ainoa geochroa* and an ITS sequence of that species from Slovakia, deposited as *Trapelia geochroa* (AF274078). The next set of sequences deposited in Genbank came from a specimen of *A. mooreana* from the Czech Republic (AY212828 and others; Schmitt et al. 2003). We recover these sequences in Baeomycetaceae with strong support. The classification of the genus has however become confused by the introduction of DNA sequences under the name *Ainoa geochroa* that are recovered in disparate parts of Ostropomycetidae. These were uploaded to Genbank by Lumbsch et al. (2007a, 2007b; number DQ871006 and other loci) based on a specimen collected in Ecuador. We have since studied the specimen (Z. Palice 8600, F!) and determined that it is not *Ainoa geochroa*, but rather *Trapeliopsis subconcolor*, previously known from South America from Colombia and Venezuela (Hertel 1977, 1981).
  We sequenced eight loci from another set of *Ainoa mooreana* from Japan and three from *Trapeliopsis subconcolor* from China, and are now able to triangulate. The Japanese *A. mooreana* fully matches the Czech specimen and its ITS is 100 % identical to the original, Slovakian ITS sequence of *A. geochroa* published by Lumbsch et al. (2001b). The sequences from the Ecuadorian specimen are however heterogeneous and doubtfully originate from the same fungus. The nuLSU sequence (DQ871006) differs from *Dibaeis baeomyces* (AF279385) by only 15 nucleotide positions and most probably belongs to an unknown Icmadophilaceae. Excluding nuLSU, the sequences of mtSSU and RPB1 from the Ecuadorian specimen place it in Baeomycetaceae in a monophyletic group with *Trapeliopsis subconcolor* from China, in agreement with its morphology and chemistry. Furthermore, the Ecuadorian nuLSU sequence does not match Chinese *T. subconcolor* nuLSU; we included both sequences in the nuLSU gene tree (Online Resource 5c) to demonstrate this point. We suspect that a mix-up occurred with the nuLSU sequence of the Ecuadorian specimen and recommend that this sequence (DQ871006) be flagged as a likely error in Genbank. As for *Trapeliopsis subconcolor*, we propose that this species, which is not closely related to *Trapeliopsis*, be treated in a new genus (see Taxonomic changes).
3. *Trapeliopsis aeneofusca* and *T. gelatinosa* are mutually paraphyletic and can be considered synonyms (the older name is *T. gelatinosa* Flörke 1809). Purvis and Smith (2009) already suspected that *T. aeneofusca* is only a pigment-deficient morph of *T. gelatinosa*.
4. Our phylogeny confirms a close relationship between *Trapeliopsis glaucopholis*, *T. steppica* and *T. wallrothii* as suspected by McCune et al. (2002; see also Printzen and McCune 2004). We refrain from recommending synonymization of these taxa as they may represent incipient speciation, but our results clearly highlight the need for work on species delimitation in this group. In this context, the recently described *T. gymnidiata* from the Canary Islands (Aptroot and Schumm 2012) and Madeira (C. Printzen, pers. obs.) also merits attention. *T. wallrothii* is the oldest name in the complex and also the type of the genus (Hafellner 1984).
5. We recover *Trapeliopsis colensoi*, *T. haumanii*, *T. glaucolepidea* and *T. precrenata* as part of a single highly supported species complex, a close relationship noted already by Galloway (2007). The separation of the mainly Southern Hemisphere *T. colensoi* from the widespread *T. glaucolepidea* is weakly supported by our phylogeny but our study was not designed to test whether they should be maintained as distinct. The similarity of *T. glaucolepidea* and *T. percrenata* has been noted in the past (e.g., Coppins and James 1984) and their distinctness questioned (Purvis and Smith 2009). We found them mutually paraphyletic and consider them synonyms, echoing the results of Palice and Printzen (2004).

### What we can and cannot say with our data

Our study includes more loci and specifically more protein-coding sequences than any previous study of the group (protein-coding loci constitute 38 % of our sequence data as opposed to 22 % for Ostropomycetidae in e.g., Miadłikowska et al. 2014). A reality of Sanger sequencing in non-model organisms is that it is impossible to obtain a full sequence sample for every isolate extracted, especially in poorly known taxonomic groups with trace amounts of DNA. This inevitably leads to a “long tail” of isolates for which partial sequence data are available. Where these isolates are from species for which other, complete sequence sets are available, we have excluded them. The practice of including taxa with large amounts of missing data and their effect on the accuracy of phylogenetic reconstructions is still under debate (for a summary see Wiens and Morrill 2011). Evidence from simulation and empirical studies shows that the impact of missing data is dependent on the phylogenetic method used and correlates with the number of characters (Wiens 2003; Dunn et al. 2003; Wiens and Morrill 2011). A consensus has emerged that distance-based phylogenetic methods (e.g., neighbour joining) and small character numbers (under 500 nucleotide positions; see Wiens and Morrill 2011) may contribute to inaccuracies in phylogenetic estimations. Even so, maximum likelihood and Bayesian phylogenetic reconstructions have been shown to be robust even in the presence of large amounts of missing data (up to 90 %; Wiens 2003) when the overall number of characters is large enough (e.g., 2000; Wiens and Moen 2008). Since our own dataset (total length of used alignment: 8978) substantially exceeds the total number of nucleotides proposed by Wiens and Moen (2008) and our data inclusion threshold for taxa with missing loci leads to concatenated sequences well above 500 nucleotides we included them in our dataset. This is consistent with evidence showing that such sequences can not only be placed accurately but also make a net positive contribution to phylogenetic analyses (Wiens and Tiu 2012; Jiang et al. 2014).

The practical interpretation of our results extends to two areas. The first of these concerns evolutionary relationships, regardless of how they are named. A legitimate question is that if our study explicitly rules out topologies acquired in past studies, how can we be sure that our own topology is not overtaken by more sampling? Our approach to this is to restrict our interpretation to relationships that are supported and/or for which alternative hypotheses can be rejected. The limitations of this approach are that it is easier to identify and reject unsupported hypotheses than propose practical resolutions for relationships that continue to be unresolved. This is exemplified by the way our data can be mined to reject or not reject sister group relationships of the trapelioids. Miadłikowska et al. (2014) argued that groups such as Arctomiales and Hymeneliales, though themselves poorly supported in their analyses, deserve recognition because they are “flanked” by well supported monophyletic groups. This explicitly assumes that the “order of divergence” of clades (“following the evolutionary split of Baeomycetales and preceding the split of Trapeliales”) is significant, despite lack of statistical support at the corresponding nodes. Our testing of the Miadłikowska et al. (2014) “order of divergence” hypothesis shows it is not only unsupported but in fact impossible to obtain given our data set (*p* = 0). Similarly we could reject other “sister group, but unsupported” relationships, such as that of trapelioids to Ostropales, which have been obtained several times in the literature. What we cannot do given our current data, however, is establish the sister group relationship of trapelioids with certainty. Several lines of evidence suggest molecular data will ultimately establish a statistically sound link between trapelioids and one or more of the three families in the BAH clade (see Results).

The second area affected by our results is how to name the orders of Ostropomycetidae. The “order of divergence” mentioned above was also inferred to be taxonomically consequential and led to erection of two orders, which under recent classification schemes makes for no fewer than five orders in Ostropomycetidae that consist of only a single family each (Arctomiales, Arctomiaceae; Baeomycetales, Baeomycetaceae; Hymeneliales, Hymeneliaceae; Sarrameanales, Sarrameanaceae; and Trapeliales, Trapeliaceae). No universal rules stipulate what constitutes an order, and there is no single correct solution. Nor is a solution necessary; the Code of Nomenclature explicitly provides for taxa of uncertain position (Art. 3.1, note 1; McNeill et al. 2012). However, we note that we recover a topological hypothesis in which Arctomiaceae, Baeomycetaceae and Hymeneliaceae form a single clade, an hypothesis that at the same time is unsupported and cannot be rejected with our data. All of these have been recently recognized as orders in their own right. We could, theoretically, adopt the name Trapeliales for the trapelioids (Trapeliaceae/Xylographaceae) as proposed by Hodkinson and Lendemer (2011), as it constitutes a third major monophyletic group in Ostropomycetidae following Ostropales and Pertusariales. The alternative hypothesis, that trapelioids from a natural phylogenetic group with the BAH clade, cannot be rejected using our data. Because no other hypothesis receives greater support, we adopt a broad view of the unresolved relationships in this sector of Ostropomycetidae and for practical reasons will treat all five families as Baeomycetales *s.lat*.. We note that this solution is similar to those of two earlier phylogenies (Wedin et al. 2005; Lumbsch et al. 2007a, b), and broadly consistent with long-running anatomy-based hypotheses (e.g., Hertel 1970; Lumbsch et al. 1995; Lumbsch 1997). A similar problem, though with fewer possible solutions, is whether to subsume the family Rimulariaceae (Hertel and Rambold 1990), typified through *Rimularia* s.str., into Trapeliaceae. We know little about what additional diversity may be uncovered in the clade we here call *Rimularia* s.str., and currently the genus is reciprocally monophyletic to the rest of Trapeliaceae. A family Rimulariaceae would have no apomorphies following the removal of *Lambiella* and *Lithographa*, which were originally included in it (Hertel and Rambold 1990), and we see little reason to maintain it as distinct from Trapeliaceae.

Another area that could affect future phylogenies is taxon sampling. Changes in taxon sampling can mediate large changes in inference, especially when the added taxa represent evolutionary “missing links” (Wiens and Tiu 2012). Specific to resolving the evolutionary relationships of trapelioid fungi, we were unable to sample several genera and species groups that may contribute to resolving future trees. Both *Amylora* (Rambold 1994) and *Coppinsia* (Lumbsch and Heibel 1998) are currently placed in Trapeliaceae (Lumbsch and Huhndorf 2010) but we could not obtain fresh material for sequencing. Ascus anatomy and thalline amyloidy suggest that *Amylora* in particular may represent a link between Hymeneliaceae and the Trapeliaceae/Xylographaceae clade; Rambold (1994) noted similarities to *Rimularia* in the broad sense. Another group of interest is the recently described genus *Cameronia*, the two species of which have muriform ascospores (Kantvilas 2012). We examined *Cameronia* ITS and a short fragment of mtSSU from a previous study (Lumbsch et al. 2012) but ultimately excluded it from our final analysis because it was below our data inclusion threshold. However, our initial results suggest *Cameronia* is related to Hymeneliaceae (data not shown). Another group that needs better sampling is *Lithographa*. This genus is heterogeneous as currently circumscribed and also includes two species with (sub)muriform ascospores (Fryday and Coppins 2007). However, many of the unsampled species of *Lithographa* occur in remote regions of the southern hemisphere. Finally, the origins of the cyanobacterium-associated Arctomiaceae will be especially interesting to clarify given the rare nature of this symbiosis in Ostropomycetidae, and the discovery of disparate lineages found to belong there (Otálora and Wedin 2013; Spribille and Muggia 2013).

### Taxonomic changes: new synonyms and combinations

*Baeomycetaceae* Dumort., *Anal. fam. pl*. (Tournay): 71 (1829) (MB80510)

= *Anamylopsoraceae* Lumbsch & Lunke in Lumbsch et al., *Pl. Syst. Evol*. 198: 285 (1995), syn. nov. (MB81979)

*Trapeliaceae* M. Choisy ex Hertel, Deutsche Bot. Ges., N.F. 4: 181 (1970) (MB81480)

= *Rimulariaceae* Hafellner, Nova Hedw. Beih. 79: 331 (1984), syn. nov. (MB81354)

*Xylographaceae* Tuck., Synopsis N. Am. Lich. Part II: 110 (1888) (MB81529)

= *Lithographaceae* Poelt, Ahmadjian & Hale, *The Lichens*: 626 (1974), *nom. inval*. Articles 36.1, 39.1 (MB81651)

The relationship between *Lambiella*, *Lithographa*, *Ptychographa* and *Xylographa* repeats the pattern recovered by Spribille et al. (2014); our present sampling further shows a highly supported sister group relationship of core genera of Trapeliaceae. In his original circumscription of Xylographaceae, Tuckerman (1888, as the family “Xylographei”) included the genera *Agyrium* and *Xylographa* as united by an “innate” (immersed) thallus and rounded to lirellate fruiting bodies that are pale to blackening (Tuckerman 1888). Watson (1929) interpreted the family to include *Lithographa*, *Ptychographa* and *Encephalographa* (now recognized as an Arthoniomycete) and dropped the inclusion of *Agyrium*. The family has otherwise seldom been used. We propose resurrecting the family Xylographaceae as distinct from Trapeliaceae to accommodate the genera *Lambiella*, *Lithographa* and *Ptychographa* and *Xylographa* (*Agyrium* has been established to be not closely related, Lumbsch et al. 2007a). Hertel (1970) already recognized the differences of the thick walled ascus with a well developed tholus of *Xylographa* and that of Trapeliaceae concluding that no close relationship exists between these groups. Here we show that Xylographaceae also includes round to broadly angular fruiting bodies (*Lambiella*). Our phylogenetic results further allow us to reject the proposal by Poelt (1974) that lirellate species with carbonized excipula, which he called Lithographaceae, are isolated from the lirellate genus *Xylographa* (which he placed in Agyriaceae).

*The new genus* Parainoa

As discussed above, *Trapeliopsis subconcolor* is more closely related to *Ainoa* than to *Trapeliopsis*, but it also does not cluster with *Ainoa* and differs from the latter in the production of depsidones, rather than tridepsides, as secondary metabolites. This species has been classified in both *Trapelia* (Hertel 1973) and *Trapeliopsis* (Hertel 1981) and the latter classification, and its relatedness to the east Asian *T. hainanensis*, was last considered to be beyond doubt (Hertel 1981). Though described from northern Italy (Anzi 1862), recent material of *T. subconcolor* has not been reported to our knowledge from Europe and the species appears to have two centres of distribution in south and east Asia and the Neotropics (Hertel 1977). *T. subconcolor* was compared to *Ainoa* (as *Trapelia mooreana*) by Hertel (1977) and differs in its creamish white papillate thallus (orangish and smooth in *Ainoa*), the more yellowish hypothecium, the conglutinated paraphyses and presence of stictic acid in the thallus, as opposed to gyrophoric acid and associated substances in *Ainoa* species (Hertel and Leuckert 1969, as *Trapelia torellii*). In *T. subconcolor*, gyrophoric acid may be present or absent in the ascomata (Hertel 1977). An SOWH test rejects the hypothesis of monophyly of *Ainoa* and *T. subconcolor* with *p* = 0 (we did not test for monophyly of *Trapeliopsis subconcolor* with *Trapeliopsis* because our topology leaves little room for that hypothesis). We refrain from combining the above-mentioned *T. hainanensis* into *Parainoa* at this time as study of two isotypes (Hertel, Lecideaceae Exs. 59, GZU!, PRA!) shows an exciple structure of tightly interwoven hyphae reaching almost to the surface of the exciple, and the presence of an incipient “stalk” in the hypothecium, recalling *Baeomyces*. The species needs further study and preferably also DNA work to compare it to *Baeomyces* s.lat. but we doubt it is congeneric with *Parainoa subconcolor*.

*Parainoa* Resl & T. Sprib., gen. nov. (MB810870)

Similar to *Ainoa* but differing in containing depsidones, similar to *Baeomyces* but differing in the complete lack of a differentiated, extended hypothecial stalk for the ascoma.

*Typus generis*: *Parainoa subconcolor* (Anzi) Resl & T. Sprib., comb. nov. (MB810871). Basionym: *Biatora subconcolor* Anzi, *Comm. Soc. Crittogam. Ital*. 1(3): 151 (1862). Type: ITALY. Prov. Sondrio, in castanetis inter pagum Rodolo et prata della Corna, Anzi, Lich. Langob. 163 (M, lectotype, FH, isolectotype, studied by Hertel 1977). Thin layer chromatography revealed stictic acid in four specimens (Arnold Lich. Exs. 938, GZU; Palice 8354, F; Palice 8600, F; and Aptroot 55,969, PRA).

Etymology: a nod to its past confusion with and occurrence near *Ainoa* in our phylogenetic hypothesis; also with reference to the problematic specimen of *P. subconcolor* from the Andean Páramo that was long confused with *Ainoa* (see Discussion).

*New combinations in* Lambiella

With the following new combinations *Lambiella* contains 10 species compared to four confirmed for *Rimularia* using molecular methods. *A posteriori* analysis confirms some differences between *Rimularia* s.str. and *Lambiella*, notably the development of depsidones in *Lambiella*. Ascus apical apparatus may also differ between the two groups of species, with all species of *Rimularia* s.str. developing a thin, vertical, non-amyloid tube that is absent in *Lambiella* species (Hertel and Rambold 1990). Although both genera occur on bare acidic rock, *Lambiella* so far accounts for all cases of occurrence on other substrates. We acknowledge our present analysis leaves 18 mostly southern hemisphere species of *Rimularia* s.lat. in limbo, but the status of most of these species cannot be easily resolved without a detailed taxonomic study of the entire group and acquisition of fresh material, often of very rare species. The task is complicated by the possibility that several species, especially *R. subconcava* from Central Asia and *R. michoacanensis* from Mexico (Timdal 2002), may not belong to either clade in the strict sense.

*Lambiella caeca* (J. Lowe) Resl & T. Sprib., comb. nov. (MB810862) Basionym: *Lecidea caeca* J. Lowe, Lloydia 2: 245 (1939). ≡ *Rimularia caeca* (J. Lowe) Rambold & Printzen, Mycotaxon 44: 454 (1992).

*Lambiella furvella* (Nyl. ex Mudd) M. Westb. & Resl, comb. nov. (MB810863) Basionym: *Lecidea furvella* Nyl. ex Mudd, Brit. Lich.: 207 (1861). ≡ *Rimularia furvella* (Nyl. ex Mudd) Hertel & Rambold, Mitt. Bot. Staatssamml. München 23: 391 (1987).

*Lambiella fuscosora* (Muhr & Tønsberg) M. Westb. & Resl, comb. nov. (MB810864) Basionym: *Rimularia fuscosora* Muhr & Tønsberg, Nordic J. Bot. 8: 649 (1989).

*Lambiella globulosa* (Coppins) M. Westb. & Resl, comb. nov. (MB810865) Basionym: *Rimularia globulosa* Coppins, Bibl. Lich. 78: 45 (2001).

*Lambiella gyrizans* (Nyl.) M. Westb. & Resl, comb. nov. (MB810866) Basionym: *Lecidea gyrizans* Nyl., Not. Sällsk. Fauna Fl. Fenn. Förh., n.s., 2: 231 (1861). ≡ *Rimularia gyrizans* (Nyl.) Hertel & Rambold, Bibl. Lich. 38: 173 (1990).

*Lambiella hepaticola* (Kantvilas & Coppins) Resl & T. Sprib., comb. nov. (MB810867) Basionym: *Rimularia hepaticola* Kantvilas & Coppins, Bibl. Lich. 78: 41 (2001). Recently acquired DNA sequence data (not shown) confirm that this species belongs in *Lambiella*.

*Lambiella impavida* (Th.Fr.) M. Westb. & Resl, comb. nov. (MB810868) Basionym: *Lecidea impavida* Th.Fr., Kongl. Svenska Vetensk. Acad. Handl. ser. 2, 7(2): 42 (1867). ≡ *Rimularia impavida* (Th.Fr.) Hertel & Rambold, Mitt. Bot. Staatssamml. München 23: 391 (1987).

*Lambiella sphacelata* (Th.Fr.) M. Westb. & Resl, comb. nov. (MB810869) Basionym: *Lecidea sphacelata* Th.Fr., Lichenogr. Scand. 2: 445 (1874). ≡ *Rimularia sphacelata* (Th.Fr.) Hertel & Rambold, Bibl. Lich. 38: 185 (1990).

## Electronic supplementary material

Below is the link to the electronic supplementary material.

ESM 1 (PDF 5590 kb)
