# Supplementary material for: Diagnostics for a troubled backbone: testing topological hypotheses of trapelioid lichenized fungi in a large-scale phylogeny of Ostropomycetidae (Lecanoromycetes)
Source: Fungal Divers. 2015 May 13;73:239–58. doi: 10.1007/s13225-015-0332-y (PMC4746758; doi:10.1007/s13225-015-0332-y)

Supplementary material for

Diagnostics for a troubled backbone: testing topological hypotheses of trapelioid lichenized fungi in a large-scale phylogeny of Ostropomycetidae (Lecanoromycetes)

by

Philipp Resl[1], Kevin Schneider[1], Martin Westberg[2], Christian Printzen[3], Zdeněk Palice[4], Göran Thor[5], Alan Fryday[6], Helmut Mayrhofer[1] and Toby Spribille[1,7,8]

[1]Institute of Plant Sciences, NAWI Graz, University of Graz, Holteigasse 6, A-8010 Graz, Austria

[2]Department of Botany, Swedish Museum of Natural History, P.O. Box 50007, SE-104 05 Stockholm, Sweden

[3]Senckenberg Forschungsinstitut und Naturmuseum, Senckenberganlage 25, D-60325 Frankfurt am Main, Germany

[4]Institute of Botany, Academy of Sciences of the Czech Republic, Zámek 1, 252 43 Průhonice, Czech Republic; and Charles University in Prague, Faculty of Sciences, Department of Botany, Benátská 2, 128 01 Praha 2, Czech Republic

[5]Department of Ecology, Swedish University of Agricultural Sciences, P. O. Box 7044, SE-750 07 Uppsala, Sweden

[6]Herbarium, Department of Plant Biology, Michigan State University, East Lansing, MI 48824, U.S.A.

[7]Division of Biological Sciences, University of Montana, 32 Campus Drive, Missoula, MT 59812, U.S.A.

[8]corresponding author; e-mail toby.spribille@mso.umt.edu

Online Resource 1: Accession table and overview of sequence samlping per locus and major clade

ID	Name	Voucher specimen	geographical origin	originally published	ITS+5.8S	SSU	LSU	mtSSU	MCM7	RPB1	RPB2	EF1-alpha	comments
**CP1030**	Trapeliopsis aeneofusca	Peksa s.n. (hb. Peksa)	Czech Republic: Northern Bohemia, Jizerské Hory Mts.	here	KR017051			KR017341		KR017417			
**CP1033**	Trapeliopsis colensoi	Kantvilas 19/04 (FR)	Australia: Tasmania, Cradle Mtn Lake	here	KR017052			KR017346		KR017418	KR017540		
**CP937**	Trapeliopsis aeneofusca	P. & B. v. d. Boom 31614 (hb. van den Boom)	Czech Republic: Western Bohemia, SSE of Klatovy	here	KR017143			KR017397		KR017414			
**CP940**	Trapeliopsis colensoi	Kantvilas 5/03 (FR)	Australia: Tasmania, Travellers Rest Lake	here	KR017144			KR017398	KR017695	KR017415		KR017594	
**CP942**	Trapeliopsis flexuosa	Palice 6744 & Voříšková (hb. Palice; PRA)	Czech Republic: North Bohemia	here	KR017145				KR017696	KR017416		KR017595	
**CP943**	Trapeliopsis flexuosa	Palice 8079 & O. Peksa (hb. Palice; PRA)	Czech Republic: East Bohemia	here	KR017146		KR017232	KR017399			KR017535	KR017596	
**CP945**	Trapeliopsis gelatinosa	B. Buryová 6966 (hb. Palice; PRA)	U.K.: Scotland, Perth & Kinross, c. 8 km NNE of Pitlochry town	here	KR017147			KR017400		KR017419			
**CP946**	Trapeliopsis glaucolepidea	Halda & Palice 8065 (hb. Palice; PRA)	Ireland: Co. Cork, Bantry area	here	KR017148			KR017401		KR017420	KR017536		
**CP947**	Trapeliopsis glaucolepidea	Palice 8500 (hb. Palice; PRA)	Ecuador: prov. Carchi, Volcán Chiles	here	KR017149			KR017402	KR017697	KR017421	KR017537	KR017597	
**CP948**	Trapeliopsis glaucopholis	McCune 25906 (hb. McCune)	U.S.A:, California, Monterey Co.	here			KR017233	KR017403		KR017422		KR017598	
**CP949**	Trapeliopsis granulosa	B. Buryová 6961 (hb. Palice; PRA)	U.K.: Scotland, Perth & Kinross, c. 5 km N of Pitlochry town	here	KR017150			KR017404		KR017423		KR017599	
**CP950**	Trapeliopsis haumanii	Palice 8517 (hb. Palice; PRA)	Ecuador: prov. Carchi, Volcán Chiles	here	KR017151			KR017405		KR017424	KR017538		
**CP951**	Trapeliopsis percrenata	Palice 8066 & Peksa (hb. Palice; PRA)	Czech Republic: Eastern Bohemia, Ždárské vrchy Mts.	here	KR017152			KR017406	KR017698	KR017425		KR017600	
**CP952**	Trapeliopsis percrenata	P. & B. v. d. Boom 31546 (hb. van den Boom)	Belgium	here	KR017153			KR017407	KR017699	KR017426		KR017601	
**CP954**	Trapeliopsis steppica	McCune 25909 (hb. McCune)	U.S.A.: California, Monterey Co.	here	KR017154		KR017234	KR017408		KR017427			
**CP955**	Trapeliopsis viridescens	Palice 5966 (hb. Palice; PRA)	Czech Republic: Southern Bohemia, Šumava Mts.	here	KR017155			KR017409				KR017593	
**CP956**	Trapeliopsis wallrothii	P. & B. v. d. Boom 21034 (hb. van den Boom)	Portugal: Alentejo, Mértola	here	KR017156		KR017235	KR017410	KR017700			KR017584	
**CP959**	Trapeliopsis viridescens	Tønsberg 30823 (BG)	U.S.A.: Tennessee, Sevier Co.	here				KR017411	KR017701		KR017539	KR017583	
**CP960**	Parainoa subconcolor	Aptroot 55939 (FR)	China: Yunnan, Jianchuan Co., Mt. Shibaoshan Park	here	KR017133		KR017236	KR017412					
**KS18**	Trapelia coarctata	Resl 1149 (GZU)	Austria: Carinthia, Hochrindl	here	KR017066		KP794971	KR017301			KR017491	KR017611	
**KS19**	Trapelia glebulosa	Resl 1147 (GZU)	Austria: Styria, Handalm	here	KR017067		KR017166	KR017362	KR017636	KR017474	KR017492	KR017542	
**KS20**	Trapelia glebulosa	Lendemer 7253 (GZU)	U.S.A.: Ohio, Scioto Co, Shawnee State Forest	here			KR017157	KR017302				KR017612	
**KS21**	Trapelia glebulosa	Spribille s.n., 03.09.2010 (GZU)	U.S.A.: Alaska, edge of town of Skagway	here	KR017068		KR017158			KR017481	KR017493	KR017635	
**KS22**	Trapelia glebulosa	Resl 1148 (GZU)	Austria: Carinthia, Hochrindl	here	KR017069		KR017159	KR017354	KR017637	KR017436	KR017494	KR017543	
**KS23**	Trapelia coarctata	Nadyeina s.n. (GZU)	Ukraine: Donetsk upland, gully "Kriven'ky Yar"	here			KR017160	KR017303		KR017482		KR017610	
**KS24**	Trapelia coarctata	Lendemer 18687 (GZU)	U.S.A.: Pennsylvania, State Game Lands No. 209	here	KR017096		KR017161			KR017483		KR017544	
**KS28**	Trapelia obtegens	Hafellner 72498 (GZU)	Austria: Styria, Hebalpe	here	KR017070		KR017162		KR017638			KR017545	
**KS30**	Trapelia glebulosa	Hafellner 75926 (GZU)	Austria: Styria, Graditschkogel	here	KR017075		KR017163					KR017617	
**KS32**	Trapeliopsis granulosa	Resl 1150 (GZU)	Austria: Carinthia, Hochrindl	here	KR017077		KR017164	KR017304				KR017607	
**KS33**	Trapeliopsis granulosa	Lendemer 19688 (GZU)	U.S.A.: California, Tuolomne Co., Yosemite National Park	here	KR017082		KP794972	KR017305	KR017639			KR017546	
**KS34**	Trapeliopsis sp. Hansen s.n.	Hansen s.n., Lich. Groen. Exs. 1164 (GZU)	Denmark: Greenland, Jakobshavn	here	KR017083		KR017165	KR017363	KR017640	KR017433		KR017547	
**KS36**	Trapeliopsis granulosa	Obermayer 12017 (GZU)	Austria: Styria, Seetaler Alpen	here	KR017084		KR017168	KR017306					
**KS38**	Trapeliopsis pseudogranulosa	Spribille 32119 (GZU)	Slovakia: Žilinský kraj, Vyšna Boca	here	KR017071		KR017169	KR017307		KR017428			
**KS46**	Trapelia coarctata	Hafellner 78428 (GZU)	Austria: Carinthia, Saualpe	here	KR017072		KR017170	KR017353	KR017641	KR017435		KR017548	
**KS47**	Trapelia glebulosa	Hafellner 74860 (GZU)	Austria: Styria, Koralpe	here	KR017053		KR017171			KR017437		KR017549	
**KS57**	Trapelia obtegens	Hafellner 77997 (GZU)	Austria: Carinthia, Saualpe	here	KR017057		KR017172	KR017308	KR017642				
**KS61**	Trapelia coarctata	Resl 1154 (GZU)	Austria: Styria, Schöckl	here	KR017098			KR017309		KR017453		KR017606	
**KS62**	Trapelia coarctata	Hafellner 76352 (GZU)	Austria: Carinthia, Beilstein	here	KR017058		KR017173	KR017310	KR017643	KR017438		KR017550	
**KS64**	Trapelia coarctata	Hafellner 68443 (GZU)	Austria: Styria, Koralpe	here	KR017097			KR017311					
**KS65**	Trapeliopsis granulosa	Hafellner 76576 (GZU)	Austria: Styria, Mürzsteger Alpen	here	KR017059		KR017174	KR017312					
**KS66**	Trapeliopsis granulosa	Hermansson 18531 (UPS)	Russia: Komi, Ust-Tsilemsky	here	KR017060		KR017175	KR017313	KR017644	KR017434			
**KS70**	Trapeliopsis gelatinosa	Obermayer 11996 (UPS)	Austria: Styria, Gurktaler Alpen	here	KR017078			KR017314	KR017645	KR017476		KR017608	
**KS72**	Placopsis lambii	Resl 1152 (GZU)	U.S.A.: Montana, Gallatin Co., Hyalite Canyon	here	KR017076		KP794970	KR017355			KR017495	KR017628	
**KS76**	Trapeliopsis granulosa	Resl 1153 (GZU)	U.S.A.: Montana, Gallatin Co., Hyalite Canyon	here	KR017079			KR017315				KR017591	
**KS81**	Trapelia glebulosa	Spribille s.n. 09.2012 (GZU)	U.S.A.: Washington, Skamania Co., pumice plain of Mt. St. Helens	here	KR017080		KR017176	KR017316	KR017646	KR017429			
**KS83**	Trapelia glebulosa	Spribille s.n. 18.09.2011 (GZU)	U.S.A.: Montana, Flathead Co., Whitefish Range, Fitzsimmons Creek saddle	here	KR017081		KR017177	KR017356	KR017647	KR017478		KR017551	
**L547**	Schizoxylon albescens	Wedin 8235 (S)	Sweden	ITS: Muggia et al. 2011; mtSSU, RPB1, EF1: here	HQ287335	KR017239		KR017317			KR017496	KR017552	
**P104**	Trapeliopsis sp. Resl 1151	Resl 1151 (GZU)	Russia: Kemerovo Oblast, Sheregesh	here	KR017117	KR017240	KR017178	KR017318	KR017648	KR017479	KR017497	KR017553	
**P108**	Anamylopsora pulcherrima	Zhurbenko 023, 2002(GZU)	Russia: Buryatiya Republic	here	KR017064	KR017241				KR017449		KR017554	
**P109**	Trapelia macrospora	Muggia NZ-4 (2012)(GZU)	New Zealand: North Island	here	KR017102			KR017319				KR017555	
**P110**	Siphula ceratites	Spribille 38923 (GZU)	U.S.A.: Alaska, Glacier Bay National Park, Dundas Bay area	here	KR017095	KR017288	KR017179			KR017462	KR017498	KR017625	

**P116**	Placopsis clavifera	Lucy NZ-20b (2012) (GZU)	New Zealand: South Island	here	KR017054	KR017242		KR017320	KR017649		KR017499	KR017626
**P118**	Placopsis gelida	Lucy NZ-7 (2012) (GZU)	New Zealand: South Island	here	KR017055	KR017243		KR017321			KR017500	KR017627
**P122**	Agyrium rufum	Spribille s.n., 13.08.2012 (GZU)	Canada: British Columbia, Cassiar Highway, Gnat Pass	here	KR017061	KR017244	KR017180		KR017650			KR017556
**P126**	Ionaspis ventosa	Spribille 39420 (GZU)	U.S.A.: Alaska, Glacier Bay National Park, Excursion Ridge	here	KR017088		KR017181	KR017322	KR017651		KR017502	KR017613
**P127**	Ochrolechia subplicans subsp. hultenii	Spribille 38211 (GZU)	U.S.A.: Alaska, Glacier Bay National Park, Icy Straits, Fern Harbor	here	KR017121	KR017245	KR017182	KR017358				KR017557
**P128**	Baeomyces heteromorphus	Muggia NZ-12 (2012)(GZU)	New Zealand: South Island	here	KR017128	KR017246	KR017183	KR017323		KR017450	KR017503	KR017558
**P129**	Thelotrema lepadinum	Spribille 38096 (GZU)	U.S.A.: Alaska, Glacier Bay National Park, Icy Straits, Fern Harbor	here	KR017122	KR017247	KR017184	KR017324	KR017652	KR017451	KR017504	KR017585
**P131**	Fissurina insidiosa	Spribille 39035 (GZU)	U.S.A.: Alaska, Gustavus, Falls Creek	here	KR017123		KR017185	KR017325	KR017653		KR017505	KR017605
**P133**	Trapelia antarctica	Thor 28992 (UPS)	Antarctica: Dronning Maud Land	here		KR017248		KR017326	KR017654	KR017452	KR017506	KR017559
**P134**	Lithographa tesserata	Thor 27912 (UPS)	Japan: Yamanashi, Mt. Kinpu	here	KR017124		KR017186	KR017327	KR017655	KR017454	KR017507	KR017604
**P141**	Trapelia coarctata	Resl s.n. (GZU - culture collection)	Austria: Styria (cultured mycobiont)	here	KR017092	KR017249		KR017328		KR017463	KR017508	KR017560
**P142**	Gyalecta jenensis	Spribille s.n. 29.09.2012 (GZU)	Canada: British Columbia, Selkirk Mtns., Incommappleux River	here	KR017099		KR017187	KR017330	KR017656	KR017455	KR017509	KR017603
**P143**	Absconditella sp. Spribille 39168	Spribille 39168 (MSC)	U.S.A.: Alaska, Glacier Bay National Park, trail to Point Gustavus	here	KR017125	KR017250	KR017188	KR017331	KR017657	KR017456	KR017501	KR017629
**P145**	Gyalectaria diluta	Spribille 36101 (GZU)	U.S.A.: Alaska, Glacier Bay National Park, Bartlett Cove	here	KR017103	KR017251	KR017189	KR017332		KR017457	KR017510	
**P147**	Trapelia antarctica	Thor 28995 (UPS)	Antarctica: Dronning Maud Land	here	KR017056	KR017252		KR017359	KR017658	KR017458	KR017511	KR017561
**P150**	Ochrolechia sp. Spribille 38907	Spribille 38907 (GZU)	U.S.A.: Alaska, Glacier Bay National Park, Falls Creek at hydro plant	here	KR017126	KR017253	KR017190	KR017333		KR017459	KR017512	KR017616
**P164**	Stictis radiata	Spribille 36698 (GZU)	U.S.A.: Alaska, Glacier Bay National Park, Rush Point	here		KR017254		KR017334		KR017484		KR017562
**P166**	Arctomia delicatula	Spribille 37968 (GZU)	U.S.A.: Alaska, Glacier Bay National Park, West Arm, Russell Island	here		KR017255	KR017191	KR017335	KR017659	KR017488		KR017563
**P167**	Coenogonium pineti	Spribille 39097 (GZU)	U.S.A.: Alaska, Glacier Bay National Park, Bartlett Cove	here		KR017256	KR017237	KR017337	KR017660	KR017489		KR017564
**P168**	Ochrolechia frigida	Spribille 39444 (GZU)	U.S.A.: Alaska, Glacier Bay National Park, Excursion Ridge	here	KR017062	KR017257		KR017339		KR017460	KR017513	KR017565
**P171**	Thrombium epigaeum	Türk 49100 (GZU)	Austria: Carinthia	here		KR017258		KR017347	KR017661			
**P173**	Thamnolia vermicularis	Resl 1136 (GZU)	Russia: Altai Republic	here	KR017094	KR017287		KR017348	KR017662	KR017461		KR017566
**P188**	Phlyctis argena	Spribille 36464 (GZU)	U.S.A.: Alaska, Glacier Bay National Park, Seebree Island	here	KR017093			KR017349				
**P196**	Loxospora elatina	Spribille s.n., 03.08.2013 (GZU)	Austria: Upper Austria, Nationalpark Kalkalpen	here	KR017063	KR017259	KR017192	KR017350		KR017485		
**P201**	Trapelia coarctata	Thor 20045 (UPS)	Sweden: Bohuslän, Nordkoster	here	KR017073		KR017193			KR017486		
**P202**	Trapelia coarctata	Nordin 6647 (UPS)	Sweden: Jämtland, shore of lake Ånnsjön	here	KR017074		KR017194	KR017351		KR017487		KR017602
**P218**	Trapeliopsis flexuosa	Björk 14178 (hb. Björk)	U.S.A: Idaho, Q'emiln Park	here	KR017091		KR017195	KR017352				
**P78**	Baeomyces rufus	Spribille 36414 (GZU)	U.S.A.: Alaska, Glacier Bay National Park, Shag Cove	Spribille et al. 2014	KJ462264	KR017260	KJ462341	KJ462396	KR017663			KR017567
**P83**	Lambiella caeca	Printzen s.n. 2009 (FR)	U.S.A.: California, Mariposa Co., Yosemite National Park	here	KR017090		KR017196	KR017357				
**P86**	Lambiella caeca	Printzen s.n. 2009 (FR)	U.S.A.: California, Mariposa Co., Yosemite National Park	here	KR017106		KR017197	KR017360				
**P87**	Lambiella caeca	Printzen s.n. 2009 (FR)	U.S.A.: California, Tuolomne Co., Yosemite National Park	here	KR017065		KR017198	KR017329				
**P92**	Trapelia corticola	Spribille 36735 (GZU)	U.S.A.: Alaska, Glacier Bay National Park, Bartlett Cove	here			KR017199	KR017361				
**P95**	Lithographa tesserata	Fryday & Spribille 38590 (GZU)	U.S.A.: Alaska, Glacier Bay National Park, Dundas Bay area	ITS, LSU: Spribille et al. 2014; all others here	KJ462269	KR017261	KJ462346	KR017340				KR017568
**R04**	Lambiella impavida	Westberg 10-064 (S)	Sweden: Jämtland	here	KR017104		KR017200	KR017364	KR017664			
**R05**	Lambiella globulosa	Westberg 08-349 (S)	Sweden: Södermanland	here	KR017105		KR017201	KR017365	KR017665	KR017442		
**R06**	Lambiella furvella	Westberg 09-098 (S)	Sweden: Södermanland	here	KR017118		KR017202	KR017366	KR017666			
**R07**	Rimularia badioatra	Westberg s.n. (S)	Sweden: Dalsland	here	KR017116		KR017203	KR017367	KR017667			
**R08**	Rimularia limborina	Westberg 12-070 (S)	Norway: Sør-Trøndelag	here	KR017108		KR017215	KR017368	KR017668	KR017446		
**R09**	Lambiella globulosa	Westberg P109 (S)	Sweden: Torne Lappmark	here	KR017109		KR017204	KR017369	KR017669			
**R10**	Lambiella impavida	Westberg P112 (S)	Sweden: Torne Lappmark	here	KR017114		KR017205	KR017370	KR017670	KR017439		
**R11**	Lambiella impavida	Westberg P110 (S)	Sweden: Torne Lappmark	here	KR017115		KR017206	KR017371	KR017671	KR017441		
**R12**	Lambiella impavida	Westberg P111 (S)	Sweden: Torne Lappmark	here	KR017127		KR017207	KR017372	KR017672	KR017440		
**R16**	Rimularia intercedens	Westberg s.n. (S)	Austria: Carinthia	here	KR017119		KR017208	KR017373	KR017673	KR017448		
**R17**	Lambiella insularis	Westberg 09-088 (S)	Sweden: Södermanland	here	KR017100		KR017209	KR017374	KR017674			
**R18**	Lambiella insularis	Westberg 09-360 (S)	Sweden: Uppland	here	KR017101		KR017210	KR017375				
**R19**	Lambiella fuscosora	Zhdanov 223 (LE)	Russia: Krasnoyarsk kray	here	KR017130		KR017211	KR017376	KR017675			
**R20**	Lambiella gyrizans	Nordin 7014 (UPS)	Sweden: Uppland	here	KR017132		KR017212	KR017377	KR017676	KR017443		
**R21**	Rimularia intercedens	Thor 23122 (UPS)	Sweden: Södermanland	here	KR017134		KR017213	KR017336	KR017677	KR017447		
**R22**	Lambiella sphacelata	Nordin 6666 (UPS)	Sweden: Jämtland	here	KR017113		KR017214	KR017378				
**T1024**	Xylographa carneopallida	Spribille 28528 (GZU)	Canada: Yukon	Spribille et al. 2014	KJ462277		KJ462352	KJ462408				KR017587

**T1046**	Hymenelia melanocarpa	Spribille 28350-B (GZU)	Canada: Yukon, La Biche River area, top of Mt. Martin	ITS, mtSSU, LSU: Spribille et al. 2014; all others here	KJ462266		KJ462343	KJ462398	KR017678	KR017413	KR017514	
**T1050**	Xylographa trunciseda	Tonsberg 40446 (BG)	Norway: Troms	ITS, mtSSU, LSU: Spribille et al. 2014; all others here	KJ462330	KR017262	KJ462389	KJ462455	KR017679	KR017430	KR017515	KR017586
**T1053**	Xylographa hians	Spribille 36071-B (GZU)	U.S.A.: Alaska	Spribille et al. 2014	KJ462289	KR017290	KJ462361	KJ462417				KR017621
**T1062**	Rimularia limborina	Fryday 9520 (MSC)	U.S.A.: California, Mariposa Co., W of Pigeon Gulch	LSU: Spribille et al. 2014; all others here	KJ462273	KR017277	KJ462404	KJ462349	KR017680			KR017569
**T1068**	Xylographa opegraphella	Spribille s.n., 2009 (GZU)	Canada: New Brunswick	Spribille et al. 2014	KJ462292	KR017291	KJ462364	KJ462420	KR017702			KR017623
**T1072**	Lambiella caeca	Spribille 36295 (GZU)	U.S.A.: Alaska, Glacier Bay National Park, Park Entrance (Moose Meadows)	here		KR017263	KR017216	KR017338	KR017681	KR017444		KR017570
**T1073**	Lambiella caeca	Spribille s.n., 01.06.2012 (GZU)	U.S.A.: Montana, Missoula Co., Pattee Canyon	here	KR017131		KR017217	KR017379		KR017445		KR017571
**T1074**	Placopsis cribellans	Fryday s.n., 04.09.2012 (GZU)	U.S.A.: Alaska, Glacier Bay National Park, Bartlett Cove	here	KR017086		KR017218	KR017380				
**T1087**	Loxosporopsis corallifera	Spribille 39319 (GZU)	U.S.A.: Alaska, Gustavus, Falls Creek	here	KR017089	KR017264	KR017219	KR017381	KR017682		KR017516	KR017572
**T1088**	Ainoa mooreana	Nordin 7455 (UPS)	Sweden: Jämtland, Åre parish	ITS, mtSSU, LSU: Spribille et al. 2014; all others here	KJ462262	KR017265	KJ462339	KJ462394	KR017683	KR017464	KR017517	KR017573
**T1091**	Lambiella insularis	Spribille s.n., 07.09.2012 (GZU)	U.S.A.: Montana, Missoula Co., N end Salmon Lake	ITS, LSU, mtSSU :Spribille et al. 2014, SSU, MCM7, RPB1, RPB2: here	KJ462268	KR017266	KJ462345	KJ462400	KR017684	KR017477	KR017518	KR017574
**T1097**	Trapelia obtegens	Spribille 30269 (GZU)	Spain: Asturias, Muniellos Nature Reserve	here			KR017220	KR017345	KR017685		KR017519	KR017575
**T1099**	Trapelia corticola	Spribille 30032 (GZU)	U.S.A.: Idaho, Shoshone Co., Hobo Cedars	here	KR017135	KR017267		KR017382	KR017686	KR017465		KR017576
**T1116**	Rimularia gibbosa	Spribille & Wagner s.n., 26.11.2011-A (GZU)	U.S.A.: Montana, Lincoln Co., W side Lake Koocanusa just N of bridge	here	KR017111	KR017270	KR017221	KR017342	KR017688			KR017577
**T1117**	Xylographa soralifera	Spribille s.n., 2012 (GZU)	U.S.A.: Montana	Spribille et al. 2014	KJ462319	KR017292	KJ462383	KJ462443	KR017703	KR017480	KR017529	KR017624
**T1123**	Ptychographa xylographoides	Acton s.n. 30/04/2013 (GZU)	U.K.: Scotland	ITS, LSU, mtSSU: Spribille et al. 2014, SSU, MCM7, RPB1, RPB2: EF: here	KJ462272	KR017268	KJ462348	KJ462403	KR017687	KR017467	KR017520	KR017619
**T1129**	Xylographa bjoerkii	Spribille 39752 (GZU)	U.S.A: Alaska	Spribille et al. 2014	KJ462275	KR017293	KJ462351	KJ462406				KR017588
**T1133**	Xylographa pallens	Spribille s.n., 2012 (GZU)	Canada: British Columbia	ITS, mtSSU, LSU: Spribille et al. 2014; all others here	KJ462299	KR017269	KJ462369	KJ462426		KR017432	KR017521	KR017618
**T1147**	Xylographa constricta	Buck 58580 (NY)	Chile: Antártica Chilena	Spribille et al. 2014	KJ462278	KR017294	KJ462353	KJ462409		KR017473		KR017589
**T1151**	Xylographa parallela	Resl 1145 (GZU)	Austria: Carinthia	Spribille et al. 2014	KJ462304	KR017295	KJ462374	KJ462430				KR017614
**T1152**	Xylographa vitiligo	Resl 1146 (GZU)	Austria: Carinthia	Spribille et al. 2014	KJ462334	KR017296	KJ462391	KJ462458	KR017704	KR017490		KR017541
**T1264**	Rimularia badioatra	McCarthy & Pitcher s.n. 22.08.2013 (MSC)	Canada: Newfoundland, central Avalon, Clam River	here	KR017120		KR017222	KR017343	KR017689		KR017522	KR017615
**T1265**	Rimularia gibbosa	Spribille & Wagner s.n., 26.11.2011-B (GZU)	U.S.A.: Montana, Lincoln Co., W side Lake Koocanusa just N of bridge	here	KR017107	KR017271	KR017223	KR017344	KR017690		KR017523	KR017579
**T1290**	Schaereria dolodes	Spribille s.n. 05.04.2014 (GZU)	U.S.A.: Montana, Sanders Co., lower Siegel Creek	here	KR017136		KR017224	KR017383		KR017466	KR017524	KR017630
**T1291**	Schaereria fuscocinerea	Spribille s.n., 10.2013 (GZU)	Austria: Styria, Zirbitzkogel, abover Großer Winterleitensee	here	KR017085	KR017272	KR017225	KR017384	KR017691			KR017609
**T1292**	Schaereria cinereorufa	Spribille s.n., 10.2013 (GZU)	Austria: Styria, Zirbitzkogel, abover Großer Winterleitensee	here	KR017112		KR017226					
**T1293**	Rimularia gibbosa	Spribille s.n. 10.2013 (GZU)	Austria: Styria, Zirbitzkogel, near Großer Winterleitensee	here	KR017129	KR017273	KR017167	KR017387	KR017692			KR017578
**T1298**	Pertusaria pertusa	Bilovitz3636 (GZU)	Bosnia-Herzegovina: Republika Srpska, Sutjeska National Park	here	KR017137	KR017298	KP794960	KR017385	KR017706			
**T1300**	Ochrolechia subplicans subsp. Hultenii	Spribille 38350 (GZU)	U.S.A.: Alaska, Glacier Bay National Park, Excursion Ridge	here	KR017087			KR017388				
**T1323**	Trapeliopsis steppica	Spribille s.n., 2014 (GZU)	U.S.A.: Montana, Sanders Co., Clark Fork River opposite of Paradise	here			KR017238	KR017386				
**T1324**	Umbilicaria polyphylla	Spribille s.n., 2013 (GZU)	Austria: Styria, Zirbitzkogel, Großer Winterleitensee	here		KR017276	KP794976	KR017390				KR017592
**T1329**	Circinaria vagans	Resl 1155 (GZU)	Russia: Altai Republic, Kosh-Agach	here		KR017274	KP794973				KR017525	KR017580
**T1331**	Pertusaria subvelata	Spribille 39272 (GZU)	U.S.A.: Alaska, Glacier Bay National Park, near Muir Point	here	KR017110	KR017275	KR017227	KR017389				KR017581
**T162**	Xylographa lagoi	Spribille 30267 (GZU)	Spain: Asturias, Muniellos Nature Reserve	ITS, mtSSU, LSU: Spribille et al. 2014; all others here	KJ462291	KR017278	KJ462363	KJ462419	KR017693	KR017431	KR017526	KR017631
**T2402**	Xylographa septentrionalis	Spribille 25133 (CANL)	Canada: British Columbia	Spribille et al. 2014	KJ462317	KR017297	KJ462382	KJ462442	KR017705			KR017620
**T2404**	Xylographa difformis	Spribille 25050 (GZU)	Canada: British Columbia	Spribille et al. 2014	KJ462280	KR017299	KJ462354	KJ462411				KR017590
**T586**	Lambiella caeca	Spribille 20029 (GZU)	Canada: British Columbia, Goat Range, Dennis Creek	here	KR017138	KR017279	KR017228	KR017391		KR017475		KR017582
**T609**	Usnea intermedia	Obermayer 11839 (GZU)	Austria: Styria, Gurktaler Alpen	EF1-a, ITS, MCM7: Spribille et al. 2011b; other loci: here	JN009731					KR017468	KR017527	JN009702
**T611**	Pseudevernia cladonia	Spribille s.n., 2009 (GZU)	U.S.A.: New Hampshire, Grafton Co., Mt. Moosilauke	here	KR017139	KR017280		KR017392		KR017469	KR017528	KR017633
**T624**	Ramboldia cinnabarina	Spribille 21549 (GZU)	Canada: British Columbia, Selkirk Mtns., Badshot Range, Healy/Hall divide	here	KR017140	KR017281	KR017229					
**T636**	Heterodermia speciosa	Spribille 26300 (KLGO)	U.S.A.: Alaska, Klondike Gold Rush National Historical Park, Chilkoot Trail	here	KR017141	KR017282	KP794975				KR017530	KR017634
**T638**	Alectoria sarmentosa	Spribille s.n., 2009 (GZU)	Canada: British Columbia, near mouth of Halfway River on Upper Arrow Lake	EF1-a, ITS, MCM7: Spribille et al. 2011b; other loci: here	JN009706	KR017283		KR017393		KR017470	KR017531	JN009675

**T639**	Cetraria sepincola	Spribille & Wagner 32131 (GZU)	Slovakia: Nizke Tatry, between Čertovica and D'umbieř	EF1-a, ITS, MCM7: Spribille et al. 2011b; other loci: here	JN009715	KR017284		KR017394			KR017532	JN009681	
**T646**	Coccotrema hahriae	Spribille 26725 (GZU)	U.S.A.: Alaska, Klondike Gold Rush National Historical Park, Chilkoot Trail	here	KR017142	KR017285		KR017395	KR017694		KR017533	KR017632	
**T764**	Japewia subaurifera	Spribille & Wagner s.n., 2009 (GZU)	U.S.A: New Hampshire, Coos Co., ridge S of Dixville Notch	EF1-a, ITS: Spribille et al. 2011b; other loci: here	JN009716	KR017289	KR017230			KR017471	KR017534	JN009682	
**T77**	Mycoblastus sanguinarius	Spribille 30127-A (GZU)	U.S.A.: Montana, Lincoln Co., Laughing Water Creek	EF1-a, ITS, MCM7: Spribille et al. 2011a; other loci: here	JF744910	KR017286	KR017231	KR017396		KR017472		JF744823	
**T820**	Xylographa erratica	Spribille 32039 (GZU)	Russia: Khabarovskiy Krai	Spribille et al. 2014	KJ462286	KR017300	KJ462357	KJ462415	KR017707			KR017622	
**X100**	Placopsis kerguelensis	Poulsen 456 (C) (ESS-20964)	France: Kerguelen Islands	ITS, LSU: Schmitt et al. 2003; SSU, RPB1, RPB2: unpublished	AY212814	DQ366257	AY212830			DQ366255	DQ366256		
**X101**	Dibaeis baeomyces	AFTOL 358	unknown	ITS, RPB1, EF1: James et al. 2006; LSU: Bhattacharaya et al. 2000; mtSSU: Lutzoni et al. 2004; RPB2: Reeb et al. 2004	DQ782844		AF279385	AY584704		DQ842011	AY641037	DQ842008	
**X102**	Coenogonium luteum	AFTOL 352	U.S.A.	ITS: Schmull et al. 2011; SSU, LSU: Bhattacharaya et al. 2000; mtSSU: Lutzoni et al. 2004; RPB2: Reeb et al. 2004	HQ650710	AF279386	AF279387	AY584699			AY641038		
**X104**	Stictis radiata	AFTOL 398	U.S.A.: Oregon	ITS: James et al. 2006; mtSSU, RPB2: Reeb et al. 2004; LSU: Lutzoni et al. 2001	DQ782846	U20610	AF356663	AY584727			AY641079		
**X106**	Diploschistes cinereocaesius	AFTOL 328	Costa Rica: San Jose	ITS: Schmull et al. 2011; SSU, LSU, mtSSU, RPB1, RPB2: Miadlikowska et al. 2006; EF: Spatafora et al. 2006	HQ650715	DQ883790	DQ883799	DQ912306		DQ883742	DQ883755	DQ883774	
**X108**	Fissurina insidiosa	AFTOL 1662	U.S.A.: North Carolina	Miadlikowska et al. 2006		DQ973022	DQ973045	DQ972995			DQ973083		
**X110**	Aspicilia caesiocinerea	AFTOL 653	U.S.A.: North Carolina	ITS: Schmull et al. 2011; SSU, LSU, mtSSU, RPB1, RPB2: Miadlikowska et al. 2006	HQ650636	DQ986736	DQ986778	DQ986892		DQ986851	DQ992469		
**X111**	Ochrolechia juvenalis	AFTOL 374	U.S.A.: Oregon	ITS: Schmull et al. 2011; SSU, LSU, RPB2: Reeb et al. 2004	HQ650719	AY640997	AY640957				AY641055		
**X113**	Loxospora ochrophaea	AFTOL 879	U.S.A.: Maine	ITS: Schmull et al. 2011; LSU, mtSSU, RPB1, RPB2: Miadlikowska et al. 2006	HQ650641		DQ986750	DQ986900		DQ986822	DQ992434		
**X114**	Loxospora cismonica	AFTOL 878	U.S.A.: Maine	ITS: Schmull et al. 2011; SSU, LSU, mtSSU, RPB2: Miadlikowska et al. 2006	HQ650640	DQ986742		DQ986899			DQ992433		
**X115**	Aspicilia cinerea	AFTOL 647	U.S.A.: North Carolina	ITS: Schmull et al. 2011; LSU, mtSSU, RPB1, RPB2: Miadlikowska et al. 2006	HQ650637		DQ986779	DQ986890		DQ986850	DQ992468		
**X116**	Coccotrema maritimum	AFTOL 964 (Brodo 30130, CANL)	Canada: British Columbia	ITS, LSU, mtSSU: Schmitt et al. 2001; MCM7: Schmitt et al. 2010	AF329165		AF329164	AF329163	GU980991				
**X117**	Gyalectaria gyalectoides	Lumbsch 19837a (F)	Fiji	Schmitt et al. 2010			GU980983	GU980975	GU980993	GU981006			
**X118**	Coccotrema cucurbitula	Vobis s.n. (ESS-20862)	Argentina: Prov. Rio Negro	ITS, mtSSU: Schmitt et al. 2001; SSU, LSU: Lumbsch et al. 2001; MCM7: Schmitt et al. 2010; RPB1: Lumbsch et al. 2007 (Ostropo revisited)	AF329162	AF274114	AF274092	AF329161	GU980990	DQ870939			
**X119**	Coenogonium leprieurii	AFTOL 351	unknown	SSU, LSU: Kauff & Lutzoni 2002; mtSSU: Lutzoni et al. 2004; RPB2: Reeb et al. 2004		AF465457	AF465442	AY584698			AY641032		
**X120**	Diploschistes ocellatus	AFTOL 958	Spain	ITS: Martin & Winka 2000; LSU: Lumbsch et al. 2004; RPB1, RPB2, EF1: Schmitt & Lumbsch 2006 (unpublished)	AF098411	AF038877	AY605077			DQ366252	DQ366253	DQ366251	
**X121**	Thelotrema lepadinum	AFTOL 83	Austria: Styria	ITS: Schmull et al. 2011; mtSSU, RPB1, RPB2: Miadlikowska et al. 2006	HQ650717			DQ972997		DQ973067	DQ973085		
**X122**	Ionapsis lacustris	AFTOL 364 (Reeb VR 16-V-99/4, DUKE)	Canada	SSU, LSU, RPB2: Reeb et al. 2004; mtSSU: Lutzoni et al. 2004		AY640992	AY640953	AY584708			AY641047		
**X123**	Gyalecta hypoleuca	AFTOL 380	Italy	ITS: Schmull et al. 2011 ;SSU, LSU: Kauff & Lutzoni 2002; mtSSU: Fernandez-Brime et al. 2011; RPB2: Reeb et al. 2004	HQ650711	AF465460	AF465453	HQ659180			AY641060		
**X124**	Ainoa mooreana	Palice 5156 (ESS 21515)	Czech Republic	ITS, mtSSU: Schmitt et al 2001, RPB1: Lumbsch 2007 (Ostropo revisited)			AY212828	AY212850		DQ870928			
**X125**	"Ainoa geochroa" - Parainoa subconcolor	Palice 8600 (F)	Ecuador	Lumbsch et al. 2007 (Ostropo revisited)				DQ871015		DQ870927			LSU (not used:) DQ871006
**X126**	Baeomyces placophyllus	Lutzoni 97.06.29-4 (DUKE)	Canada	SSU,LSU: Lutzoni et al. 2001, RPB1: Lumbsch et al. 2007 (Ostropo revisited), RPB2: Reeb et al. 2004		AF356657	AF356658				AY641028		RPB1: not used: DQ870936 (other specimen)
**X128**	Placopsis antarctica	Poulsen 446c (C)	France: Kerguelen Islands	mtSSU: Schmitt et al 2001; RPB2: Lumbsch et al 2007 (Ostropo revisited)				AY212852			DQ870962		
**X129**	Placopsis perrugosa	AFTOL 383	Australia: Victoria	SSU, LSU: Lutzoni et al. 2001; ITS: Schmull et al. 2011; mtSSU: Lutzoni et al. 2004; RPB2: Reeb et al. 2004	HQ650709	AF356659	AF356660	AY584716		DQ870962	AY641063		
**X130**	Placopsis bicolor	Søchting 9362 (C)	France: Crozet Island	Schmitt et al. 2003	AY212816		AY212833	AY212856					
**X131**	Trapelia chiodectonioides	Becker s.n. 1994 (F)	Zimbabwe	Schmitt et al. 2003			AY212847	AY212873		DQ870999			
**X133**	Lambiella psephota	Kantvilas 335/00 (HO)	Australia	Lumbsch et al. 2007 (Ostropo revisited)			DQ871012	DQ871019		DQ870992			
**X134**	Phyllobaeis erythrella	AFTOL 329	Costa Rica: San Jose	Miadlikowska et al. 2006		DQ986734	DQ986780	DQ986888		DQ990921	DQ992471		
**X135**	Phyllobaeis imbricata	AFTOL 852	Costa Rica: San Jose	ITS: Schmull et al. 2011, SSU, LSU, mtSSU, RPB2: Miadlikowska et al. 2006	HQ650635	DQ986739	DQ986781	DQ986895			DQ992472		
**X136**	Placynthiella icmalea	S. Huhtinen 05/15 (TUR)	Finland	Stenroos et al. 2010	EU940236	EU940083	EU940160	EU940300			EU940368		

**X137**	Placynthiella uliginosa	AFTOL 1365	Finland: Uusimaa	LSU, mtSSU, RPB1, RPB2: Miadlikowska et al. 2006; ITS: Schmull et al. 2011	HQ650633		DQ986774	DQ986877		DQ986845	DQ992463	
**X138**	Placopsis pycnotheca	Søchting 7993 (C)	Chile	Schmitt et al. 2003	AY212823		AY212841	AY212864				
**X139**	Placopsis contortuplicata	Lumbsch 19012d (hb. Lumbsch)	Antarctica	Schmitt et al. 2003	AY212818		AY212835	AY212858				
**X140**	Placopsis santessonii	Messuti s.n. (hb. Lumbsch)	Chile	Schmitt et al. 2003	AY212826		AY212845	AY212858				
**X141**	Placopsis lambii	Haugan (O-L55755)	Norway	Schmitt et al. 2003	AY212819		AY212837	AY212860				
**X142**	Placopsis macrophthalma	Poulsen 227 (C)	France: Kerguelen Islands	Schmitt et al. 2003	AY212820		AY212839	AY212862				
**X143**	Trapelia coarctata	Palice & Schmitt (ESS-20966)	Czech Republic: Bohemia	mtSSU: Schmitt et al. 2003; LSU: Poulsen et al. 2001			AF274117	AY212874				
**X145**	Trapeliopsis percrenata	Vězda (Vězda Exs. 378) (hb. Lumbsch)	Czech Republic: Palice	LSU: Lumbsch & Schmitt 2001; mtSSU: Schmitt et al. 2003; RPB1: Lumbsch et al. 2007 (Ostropo revisited)			AF279302	AY212876		EF158853		
**X146**	Tremolecia atrata	Wedin 7094 (UPS)	Sweden	Wedin et al. 2005			AY853397	AY853347				
**X147**	Anamylopsora pulcherrima	Zhurbenko s.n. (ESS)	Russia: Yakutia	ITS:Lumbsch et al. 2001 (ITS sequence data), SSU: Lumbsch et al. 2001 (Agyriales)	AF274089	AF119501						
**X148**	Umbilicaria muehlenbergii	AFTOL 404	U.S.A.: Wisconsin	SSU, LSU: Reeb et al. 2004; mtSSU: Lutzoni et al. 2004; RPB1, RPB2: Miadlikowska et al. 2006		AY641016	AY640977	AY584729		DQ986842	AY641088	
**X149**	Umbilicaria arctica	AFTOL 1266 (Hestmark 04201)	Norway: Finse	ITS: Hestmark et al. 2010; SSU, LSU,mtSSU, RPB1, RPB2: Miadlikowska et al. 2006	HM161454	DQ986717	DQ986772	DQ986872		DQ986841	DQ992460	
**X150**	Umbilicaria mammulata	AFTOL 645	U.S.A.: North Carolina	ITS, LSU, RPB1, RPB2, EF1: James et al 2006; mtSSU: Miadlikowska et al. 2006	DQ782851		DQ782912	DQ912305		DQ782831	DQ782873	DQ782903
**X151**	Lasallia pustulata	AFTOL 554	Norway: Ostfold	ITS: Hestmark et al. 2010; SSU, LSU: RPB1, RPB2, EF1: Spatafora et al. 2006; mtSSU: Miadlikowska et al. 2006	HM161456	DQ883700	DQ883690	DQ986889		DQ883719	DQ883707	DQ883726
**X160**	Wawea fruticulosa	Kantvilas (F, HO)	Australia: Tasmania	Lumbsch et al. 2007 (Ostropo revisited)			DQ007347	DQ871023		DQ871005		
**X161**	Circinaria hispida	Candan 11 (ANES)	Turkey	Nordin et al. 2010			HM060760	HM060722				
**X162**	Circinaria hispida	Lumbsch (June 02, 2003) (F)	Spain	LSU, mtSSU: Schmitt et al. 2006; RPB1: Lumbsch et al. 2007 (Ostropo revisited)			DQ780305	DQ780273		DQ870933		
**X163**	Megaspora verrucosa	Nordin 6495 (UPS)	Sweden	Nordin et al. 2010			HM060725	HM060687				
**X164**	Megaspora verrucosa	Schmitt s.n. 24.05.2003 (F?)	Slovakia	LSU, mtSSU: Schmitt et al. 2006; RPB1: Lumbsch et al. 2007 (Ostropo revisited)			DQ780307	DQ780275		DQ870955		
**X165**	Lobothallia radiosa	Lumbsch s.n. (09.Aug 2004) (F)	Switzerland	LSU, mtSSU: Schmitt et al. 2006; RPB1: Lumbsch et al. 2007 (Ostropo revisited)			DQ780306	DQ780274		DQ870954		
**X166**	Ochrolechia yasudae	AFTOL 882	U.S.A.: Connecticut	Miadlikowska et al. 2006		DQ986744	DQ986776	DQ986902		DQ986848	DQ992466	
**X168**	Geoglossum nigritum	AFTOL 56	U.S.A.: Oregon	SSU, LSU, mtSSU: Lutzoni et al. 2004; RPB1, RPB2: Spatafora et al. 2006		AY544694	AY544650	AY544740		DQ471115	DQ470879	
**X169**	Peltula auriculata	AFTOL 892	Venezuela: Territorio Federal Amazonas	ITS, SSU, LSU, RPB2: James et al. 2006; mtSSU: Miadlikowska et al. 2006	DQ832329	DQ832332	DQ832330	DQ922953			DQ832331	
**X170**	Acarospora laqueata	AFTOL 1007	France: Vaucluse	SSU, LSU, RPB2: Reeb et al. 2004; RPB1: James et al. 2006		AY640984	AY640943			DQ782860	AY641024	
**X172**	Protothelenella sphinctrinoidella	Lumbsch 19031d (F)	Antarctica: Livingston Island	LSU, mtSSU: Schmitt et al. 2005; RPB1: Lumbsch et al. 2007			AY607735	AY607747		DQ870989		
**X173**	Protothelenella corrosa	Palice s.n.? (hb. Palice; PRA)	Czech Republic	LSU, mtSSU: Schmitt et al. 2005; RPB1: Lumbsch et al. 2007			AY607734	AY607746		DQ870988		
**X174**	Anzina carneonivea	Palice 4168 (hb. Palice; PRA)	Czech Republic	Schmitt et al. 2003			AY212829	AY212851				

Missing data per locus

	ITS	SSU	LSU	mtSSU	MCM7	RPB1	RPB2	EF
Obtained Sequences	106	62	82	112	72	78	50	95
Sequences from GB	57	26	80	71	3	31	31	10
Missing Sequences	42	117	43	22	130	96	124	100

Missing data per clade

	Pertusariales	Ostropales	Baeomycetales	Baeomycetaceae	Xylographaceae	Trapeliaceae
Maximum sequence number	256	120	1048	88	296	664
Available sequences	155	70	608	48	193	367
Missing Sequences	101	50	440	40	103	297

References for Online Resource 1

Bhattacharya D, Lutzoni F, Reeb V, et al. (2000) Widespread occurrence of spliceosomal introns in the rDNA genes of ascomycetes. Molecular Biology and Evolution 17:1971–1984

Fernández-Brime S, Llimona X, Molnar K, et al. (2011) Expansion of the Stictidaceae by the addition of the saxicolous lichen-forming genus Ingvariella. Mycologia 103:755–763. doi:10.3852/10–287

Hestmark G, Miadlikowska J, Kauff F, et al. (2011) Single origin and subsequent diversification of central Andean endemic Umbilicaria species. Mycologia 103:45–56. doi:10.3852/10–012

James TY, Kauff F, Schoch CL, et al. (2006) Reconstructing the early evolution of Fungi using a six-gene phylogeny. Nature 443:818–822. doi:10.1038/nature05110

Kauff F, Lutzoni F (2002) Phylogeny of the Gyalectales and Ostropales (Ascomycota, Fungi): among and within order relationships based on nuclear ribosomal RNA small and large subunits. Molecular Phylogenetics and Evolution 25:138–156

Lumbsch HT, Schmitt I, Döring H, Wedin M (2001a) ITS sequence data suggest variability of ascus types and support ontogenetic characters as phylogenetic discriminators in the Agyriales (Ascomycota). Mycological Research 105:265–274. doi:10.1017/S0953756201003483

Lumbsch HT, Schmitt I, Döring H, Wedin M (2001b) Molecular systematics supports the recognition of an additional order of Ascomycota: the Agyriales. Mycological Research 105:16–23. doi:10.1017/S095375620000321X

Lumbsch, H. T., Mangold, A., Lücking, R., García, M. A. & Martín, M. P. 2004. Phylogenetic position of the genera *Nadvornikia* and *Pyrgillus* (Ascomycota) based on molecular data. Acta Univ. Ups. Symb. Bot. Ups. 34:1, 9–17. Uppsala. ISBN 91-554-6025-9

Lumbsch HT, Schmitt I, Lücking R, et al. (2007) The phylogenetic placement of Ostropales within Lecanoromycetes (Ascomycota) revisited. Mycological Research 111:257–267. doi:10.1016/j.mycres.2007.01.006

Lumbsch T (2001) Utility of nuclear SSU and LSU rDNA data sets to discover the ordinal placement of the Coccotremataceae (Ascomycota). Organisms Diversity & Evolution 1:99–112. doi:10.1078/1439-6092-00008

Lutzoni F, Kauff F, Cox CJ, et al. (2004) Assembling the fungal tree of life: Progress, classification and evolution of subcellular traits. American Journal of Botany 91:1446–1480. doi:10.3732/ajb.91.10.1446

Lumbsch HT, Mangold A, Lücking R, García MA, Martín MP (2004) Phylogenetic position of the genera *Nadvornikia* and *Pyrgillus* (Ascomycota) based on molecular data Acta Univ. Ups. Symb. Bot. Ups. 34:1, 9–17. Uppsala

Lutzoni F, Pagel M, Reeb V (2001) Major fungal lineages are derived from lichen symbiotic ancestors. Nature 411:937–940. doi:10.1038/35082053

Martín MP, Winka K (2000) Alternative Methods of extracting and Amplifying Dna from lichens. The Lichenologist, 32:189–196. doi:10.1006/lich. 1999.0254

Miadlikowska J, Kauff F, Hofstetter V, et al. (2006) New insights into classification and evolution of the Lecanoromycetes (Pezizomycotina, Ascomycota) from phylogenetic analyses of three ribosomal RNA- and two protein-coding genes. Mycologia 98:1088–1103

Muggia L, Baloch E, Stabentheiner E, Grube M, Wedin M (2011) Photobiont association and genetic diversity of the optionally lichenized fungus *Schizoxylon albescens*. FEMS Microbiology Ecology 75: 255–272. doi:10.1111/j.1574-6941.2010.01002.x

Nordin A, Savić S, Tibell L (2010) Phylogeny and taxonomy of Aspicilia and Megasporaceae. Mycologia 102:1339–1349. doi:10.3852/09-266

Poulsen RS, Schmitt I, Søchting U, Lumbsch HT (2001) Molecular and morphological studies on the subantarctic genus *Orceolina* Agyriaceae. The Lichenologist 33:323–329. doi:10.1006/lich.2001.0327

Reeb V, Lutzoni F, Roux C (2004) Contribution of RPB2 to multilocus phylogenetic studies of the euascomycetes (Pezizomycotina, Fungi) with special emphasis on the lichen-forming Acarosporaceae and evolution of polyspory. Molecular Phylogenetics and Evolution 32:1036–1060. doi:10.1016/j.ympev.2004.04.012

Schmitt I (2001) Molecular data support rejection of the generic concept in the Coccotremataceae (Ascomycota). The Lichenologist 33:315–321. doi:10.1006/lich.2001.0325

Schmitt I, Fankhauser JD, Sweeney K, et al. (2010) Gyalectoid Pertusariaspecies form a sister-clade to Coccotrema(Ostropomycetidae, Ascomycota) and comprise the new lichen genus Gyalectaria. Mycology 1:75–83. doi:10.1080/21501201003631540

Schmitt I, Lumbsch HT, Sochting U (2003) Phylogeny of the lichen genus Placopsis and its allies based on Bayesian analyses of nuclear and mitochondrial sequences. Mycologia 95:827–835

Schmitt I, Mueller G, Lumbsch HT (2005) Ascoma morphology is homoplaseous and phylogenetically misleading in some pyrenocarpous lichens. Mycologia 97:362–374

Schmitt I, Yamamoto Y, Lumbsch HT (2006) Phylogeny of Pertusariales (Ascomycotina): Resurrection of Ochrolechiaceae and new circumscription of Megasporaceae. Journal of the Hattori Botanical Laboratory 100:753–764

Schmull M, Miadlikowska J, Pelzer M, et al. (2011) Phylogenetic affiliations of members of the heterogeneous lichen-forming fungi of the genus Lecidea sensu Zahlbruckner

(Lecanoromycetes, Ascomycota). Mycologia 103:983–1003. doi:10.3852/10–234

Schoch CL, Seifert KA, Huhndorf S, et al. (2012) Nuclear ribosomal internal transcribed spacer (ITS) region as a universal DNA barcode marker for Fungi. Proc Natl Acad Sci USA 109:6241–6246. doi:10.1073/pnas.1117018109

Spatafora JW, Sung G-H, Johnson D, et al. (2006) A five-gene phylogeny of Pezizomycotina. Mycologia 98:1018–1028

Spribille, T., B. Klug, H. Mayrhofer (2011a): A phylogenetic analysis of the boreal lichen *Mycoblastus sanguinarius* (Mycoblastaceae, lichenized Ascomycota) reveals cryptic clades correlated with fatty acid profiles. Molecular Phylogenetics and Evolution 59: 603–614

Spribille T, Goffinet B, Klug B, Muggia L, Obermayer W, Mayrhofer H (2011b) Molecular support for the recognition of the *Mycoblastus fucatus* group as the new genus *Violella* (Tephromelataceae, Lecanorales). The Lichenologist 43:445–466

Spribille T, Resl P, Ahti T, PérezOrtega S, Tønsberg T, Mayrhofer H, Lumbsch HT (2014) Molecular systematics of the wood-inhabiting, lichen-forming genus *Xylographa* (Baeomycetales, Ostropomycetidae) with eight new species. Symbolae Botanicae Upsalienses (in press)

Stenroos S, Laukka T, Huhtinen S, et al. (2009) Multiple origins of symbioses between ascomycetes and bryophytes suggested by a five-gene phylogeny. Cladistics 26:281–300. doi:10.1111/j.1096-0031.2009.00284.x

Wedin M, Wiklund E, Crewe A, et al. (2005) Phylogenetic relationships of Lecanoromycetes (Ascomycota) as revealed by analyses of mtSSU and nLSU rDNA sequence data. Mycological Research 109:159–172. doi:10.1017/S0953756204002102

Online Resource 2

Scenario A (((((Ostropales),(Arctomiaceae)),(Trapeliaceae s.lat),(Hymeneliaceae)),(Baeomycetaceae)), Rest);
Scenario B (((Trapeliaceae s.lat),(Baeomycetaceae)),Rest);
Scenario C (((Trapeliaceae s.lat),(Hymeneliaceae)),Rest);
Scenario D (((Trapeliaceae s.lat),(Ostropales)),Rest);

Online Resource 3

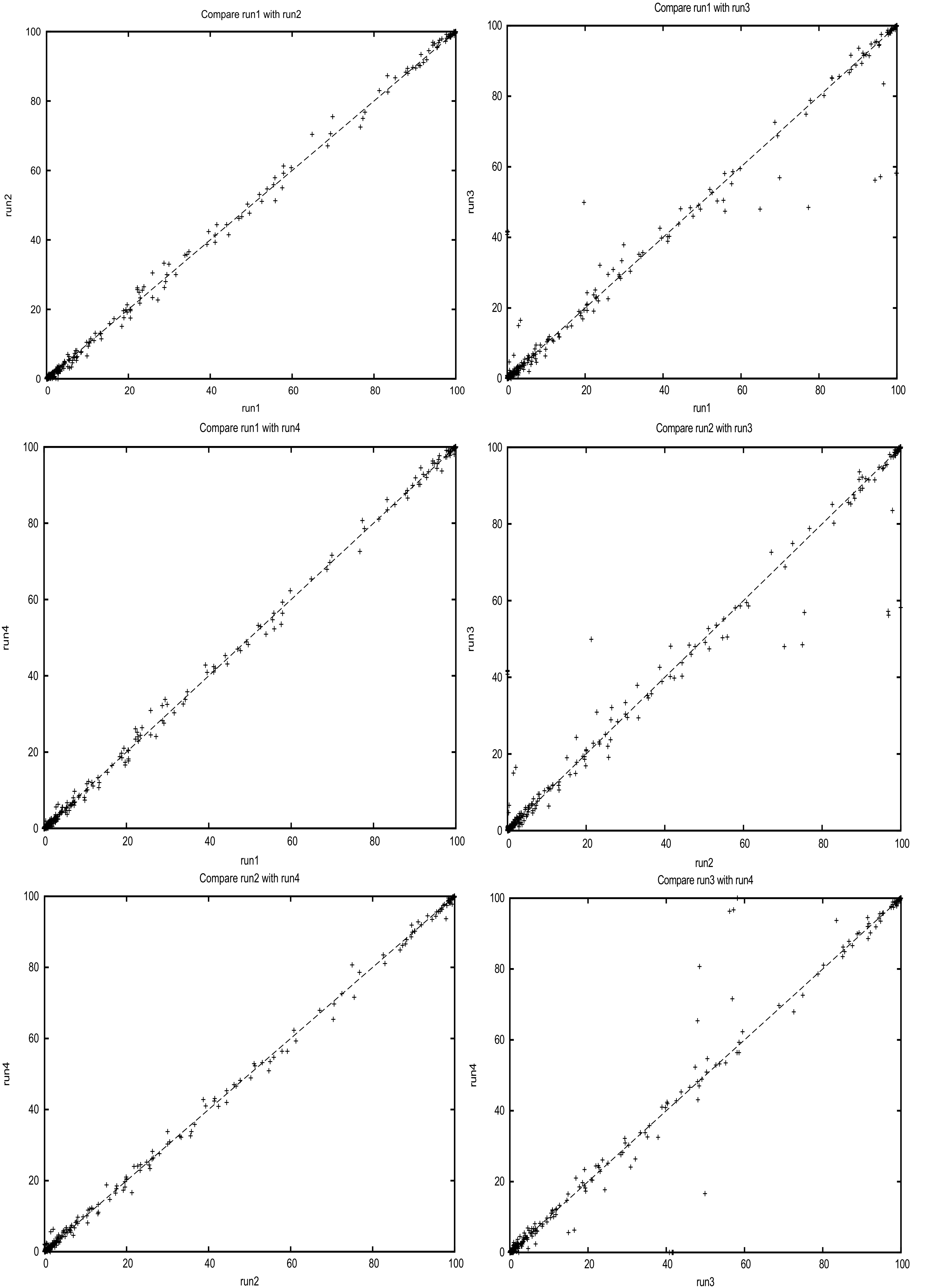

Online Resource 4

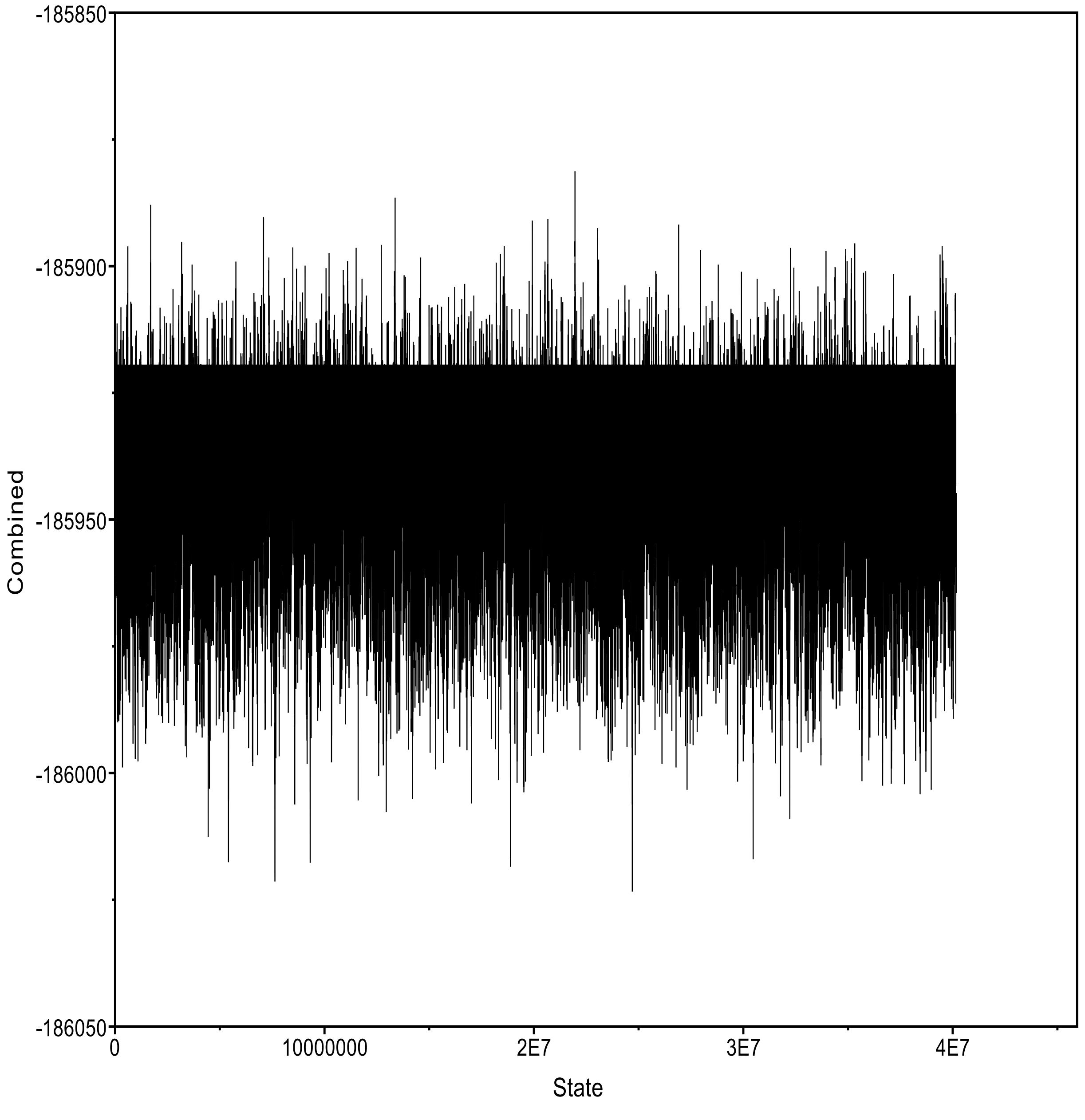

Online Resource 5a: EF gene tree

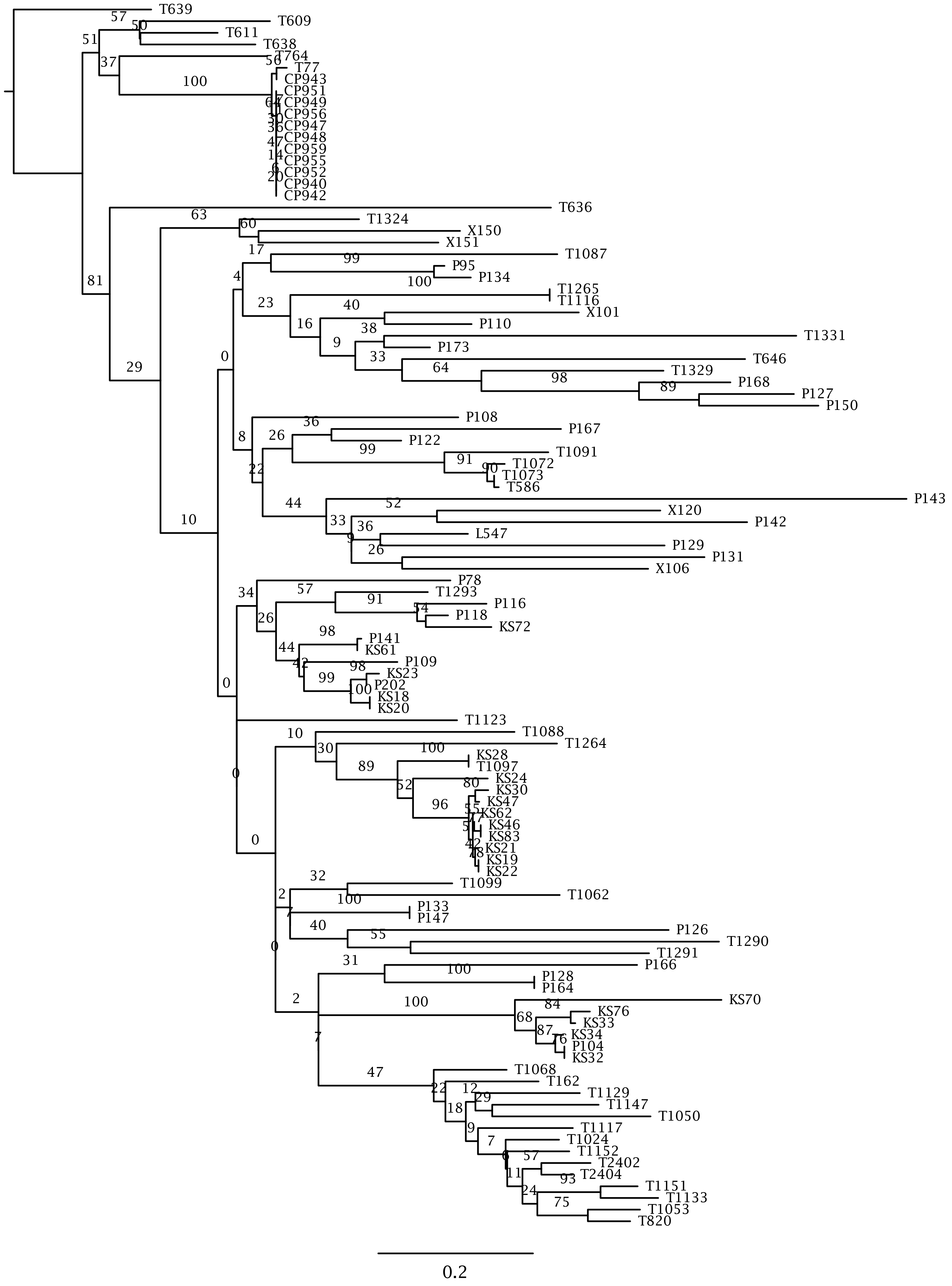

Online Resource 5b: ITS gene tree

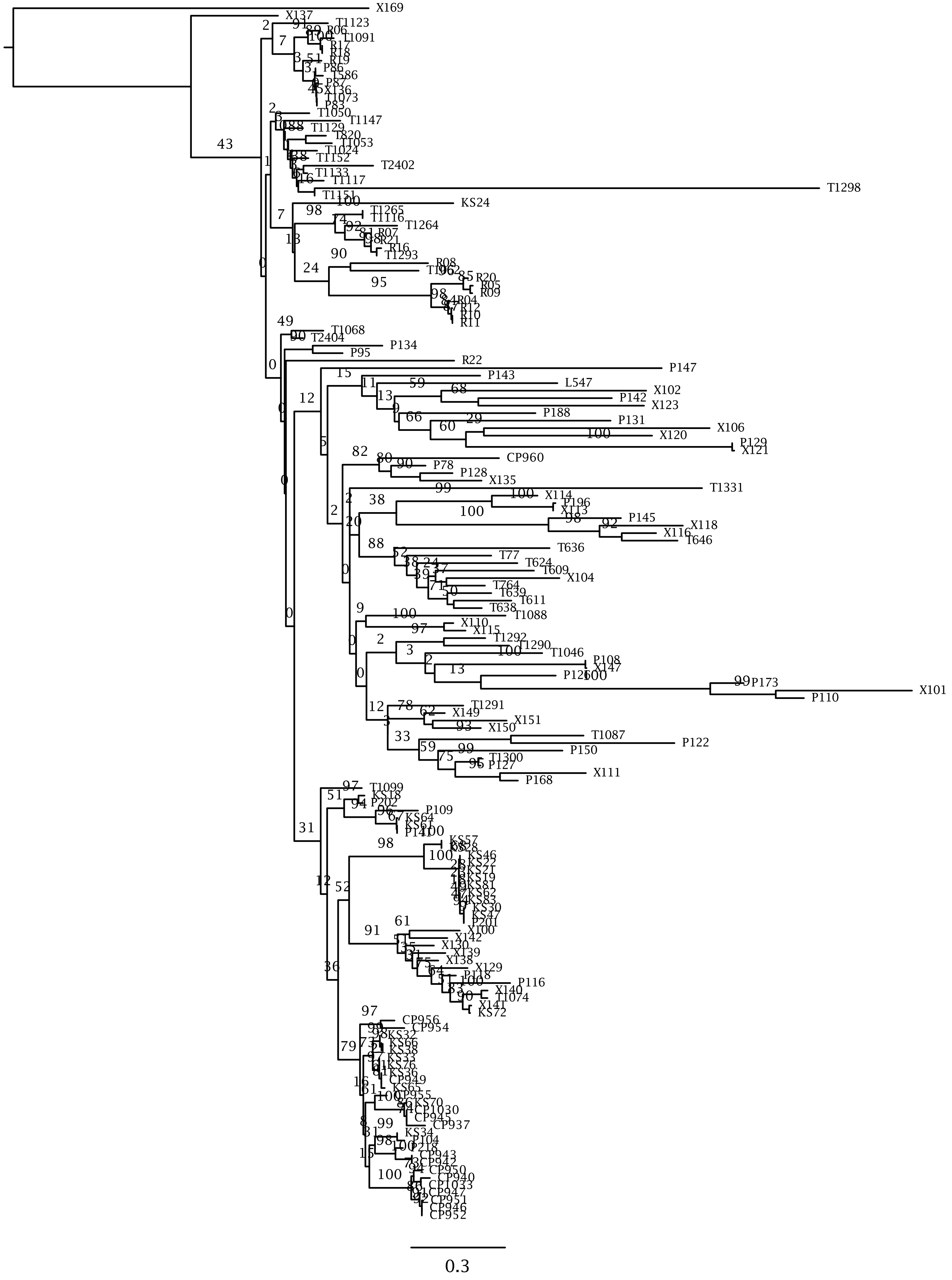

Online Resource 5c: LSU gene tree

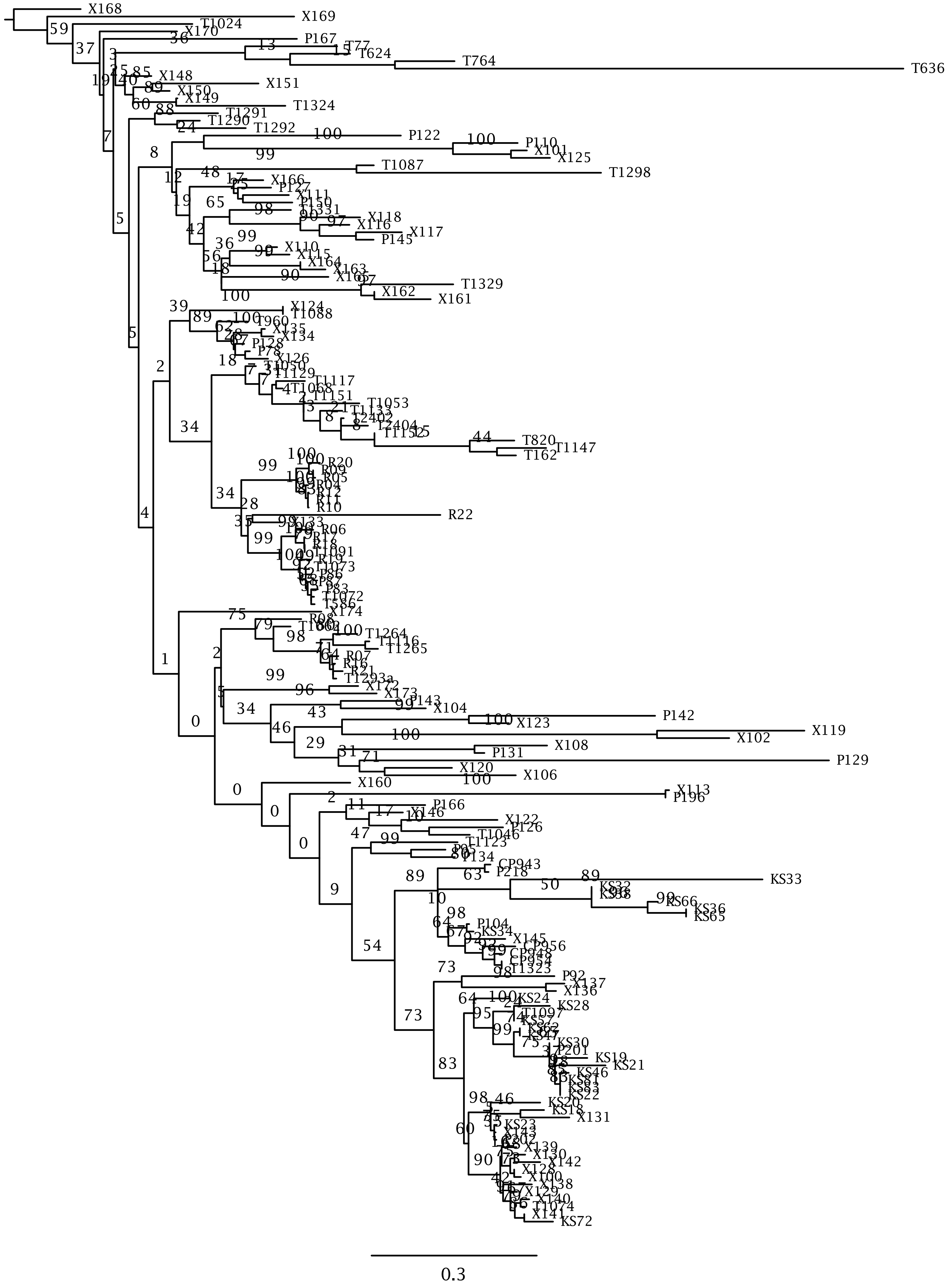

Online Resource 5d: MCM7 gene tree

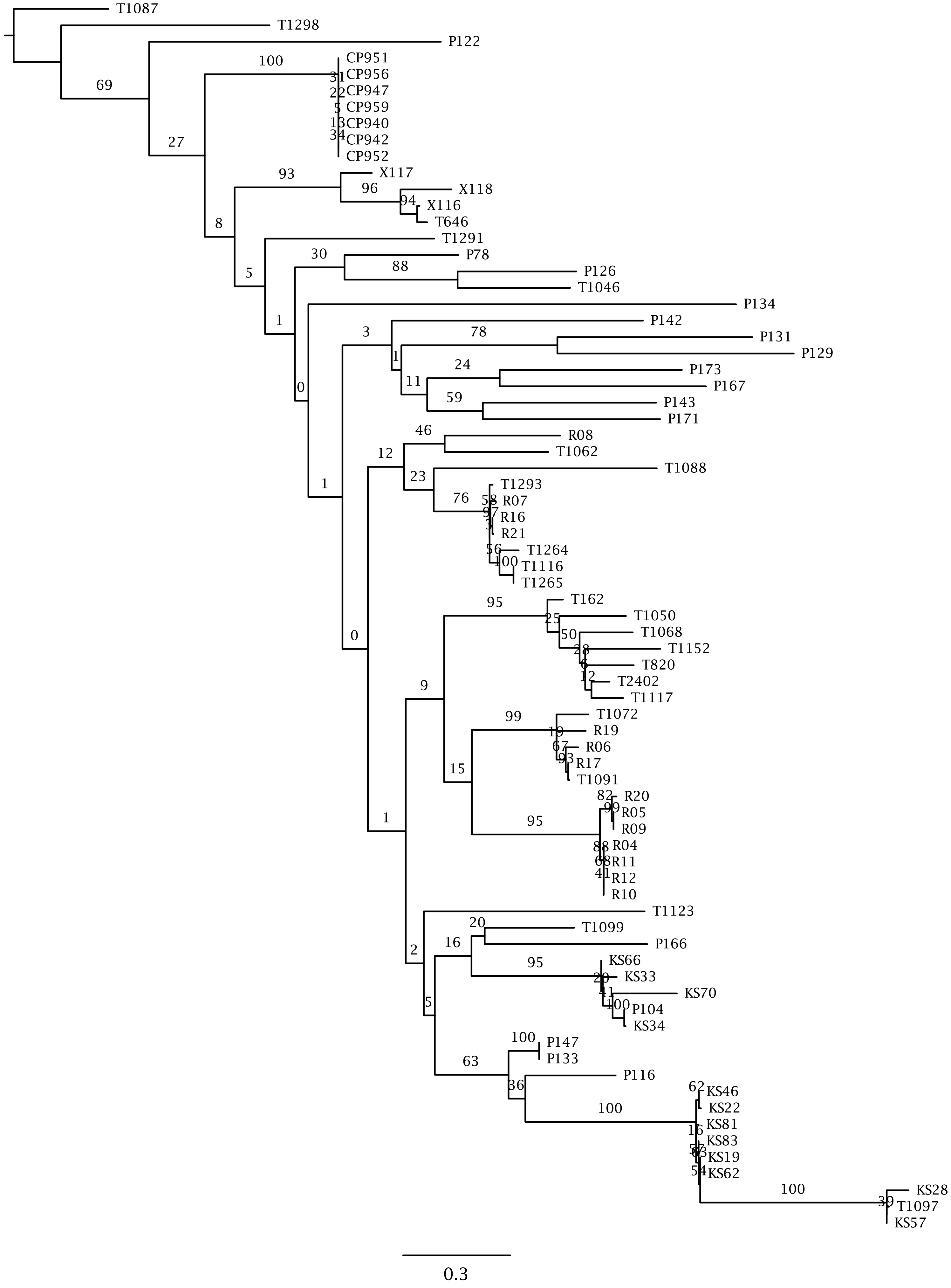

Online Resource 5e: RPB1 gene tree

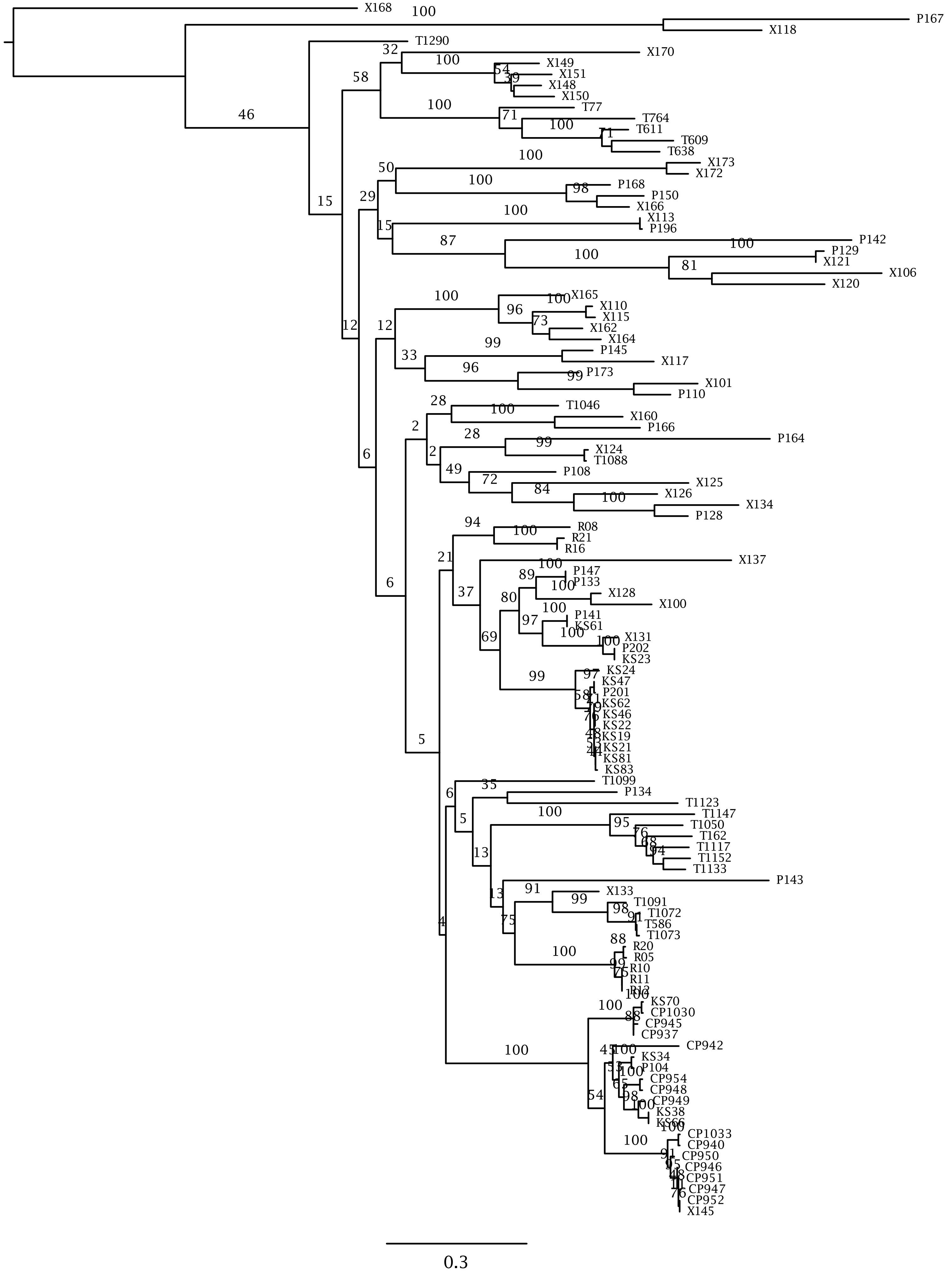

Online Resource 5f: RPB2 gene tree

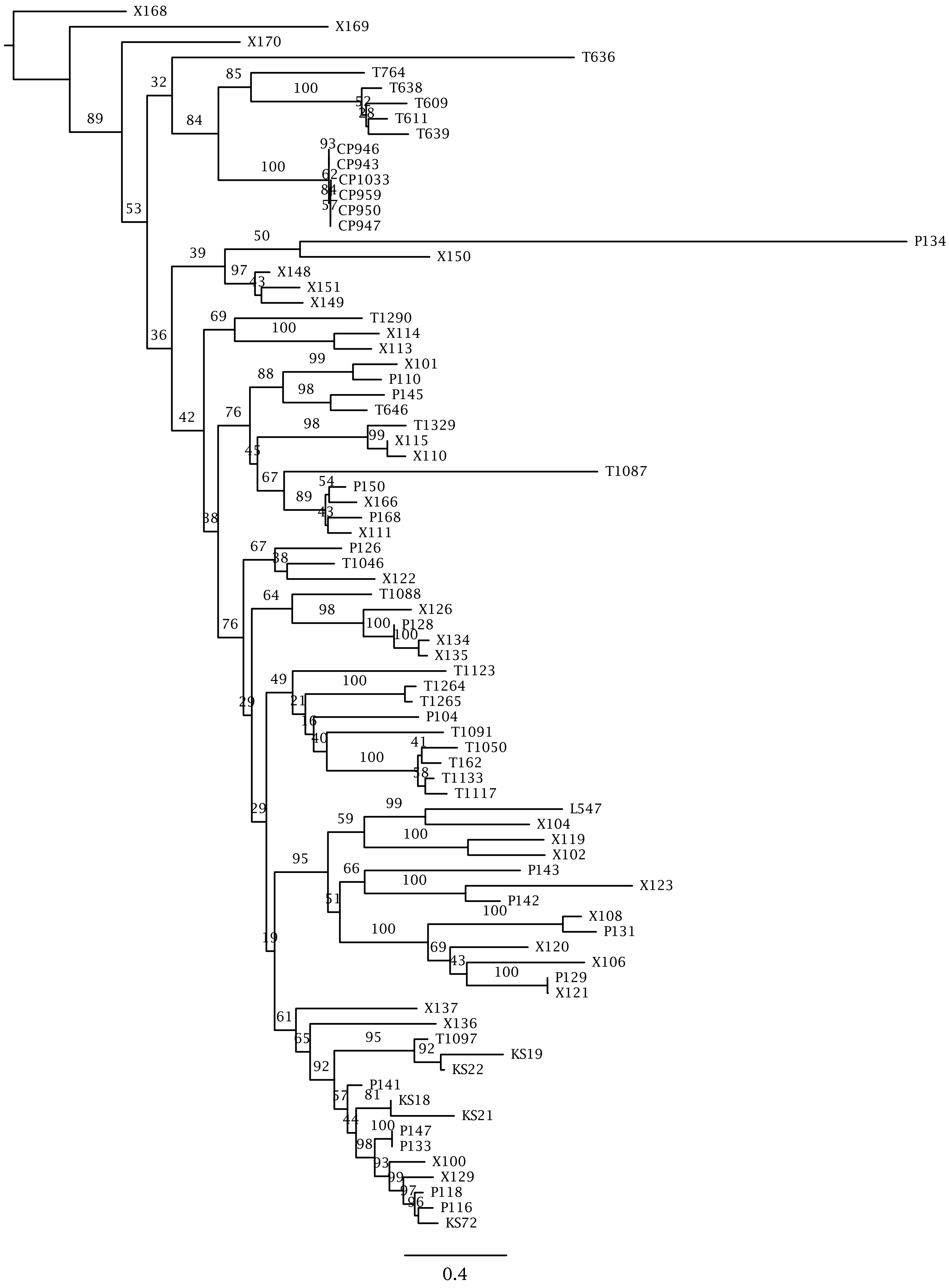

Online Resource 5g: mtSSU gene tree

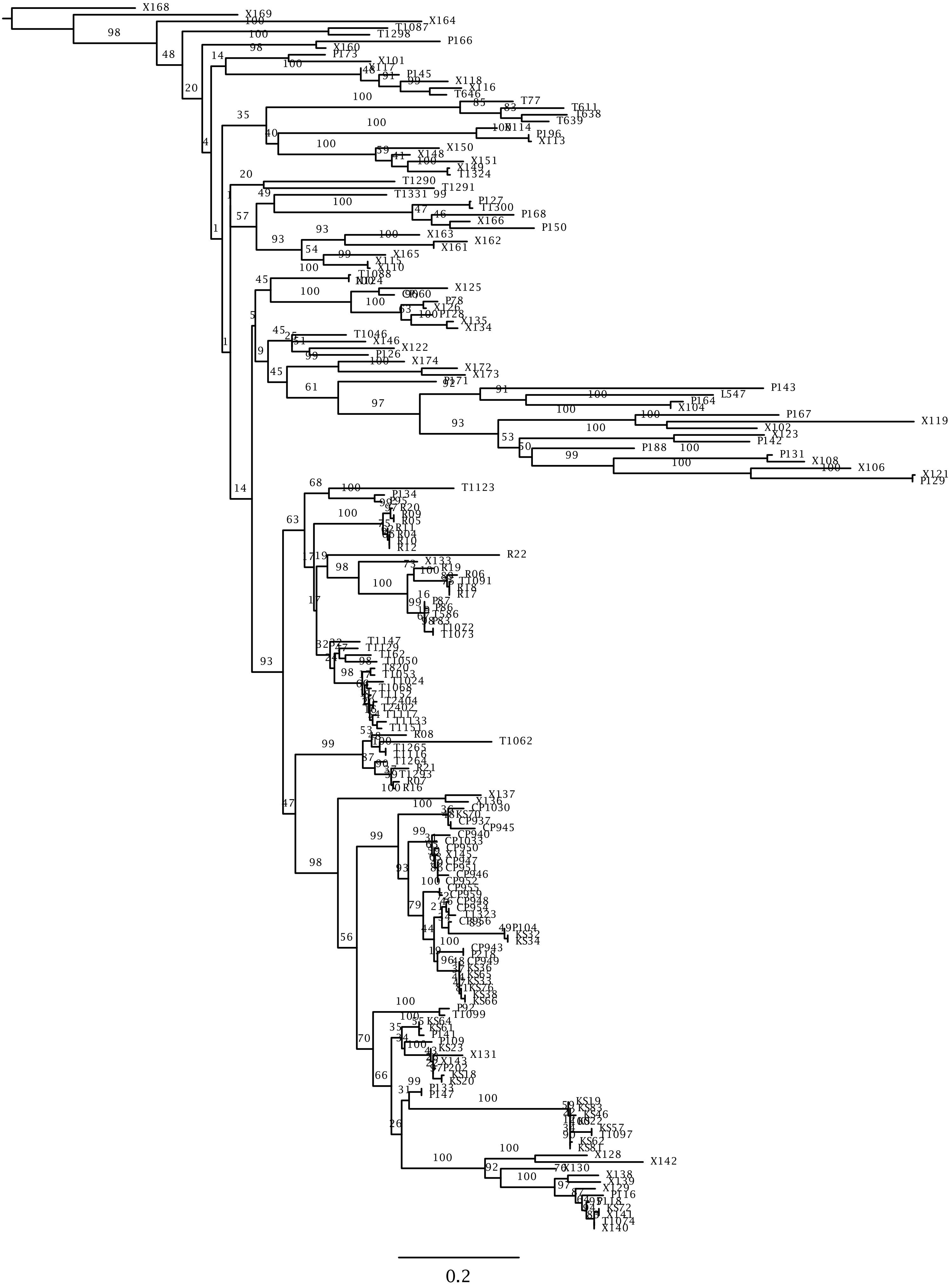

Online Resource 5h: SSU gene tree

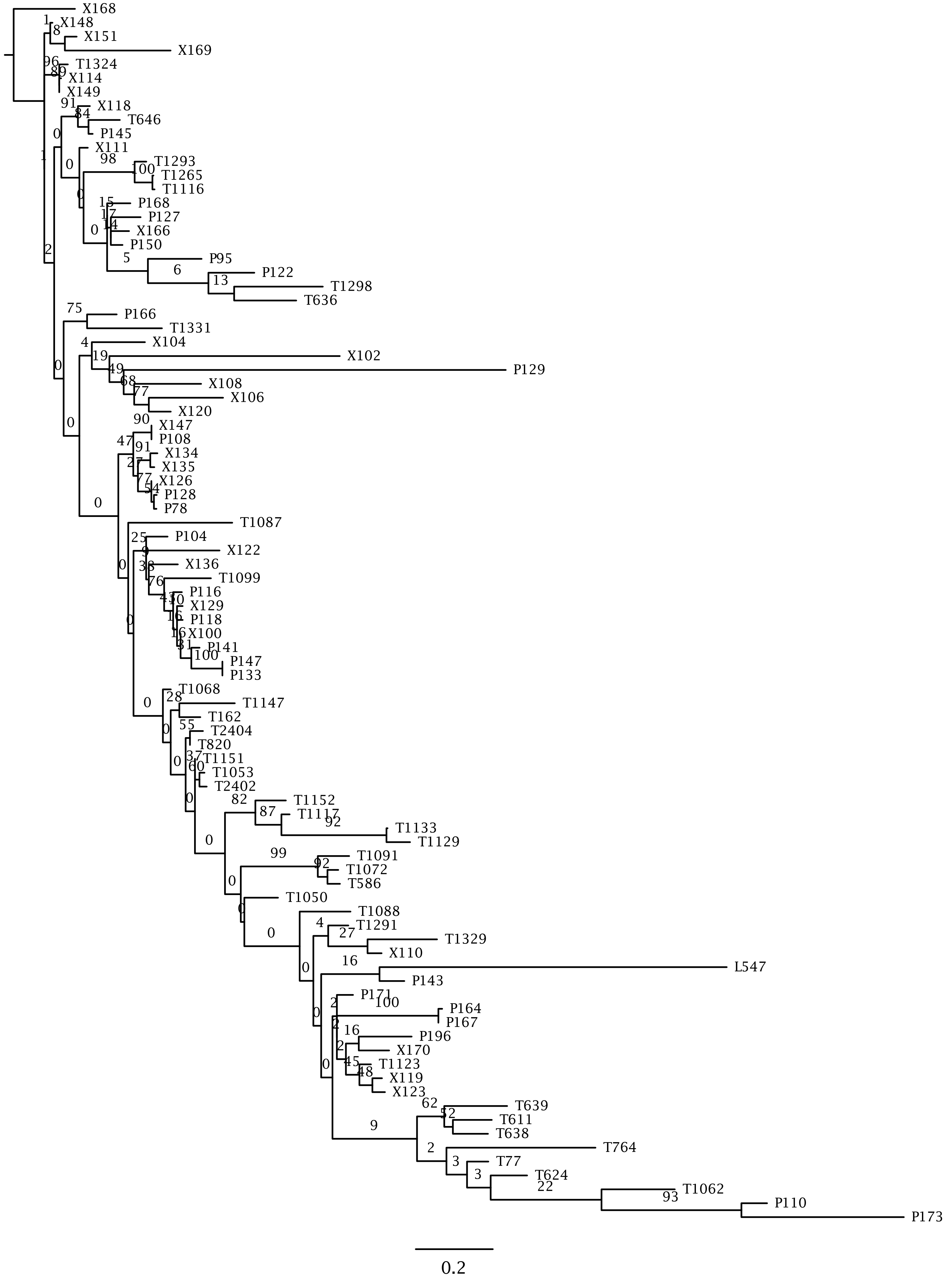

Online Resource 6

	ITS	SSU	LSU	mtSSU	MCM7	RPB1	RPB2	EF
ITS								
SSU	1							
LSU	1	0						
mtSSU	1	0	0					
MCM7	0	0	0	0				
RPB1	1	1	1	2	1			
RPB2	2	0	0	0	0	1		
EF	1	1	0	1	0	1	0	

Sequences causing conflicts according to compat.py:

ITS: X104
EF: T77, P164
RPB1: P167
mtSSU: X164

Online Resource 7

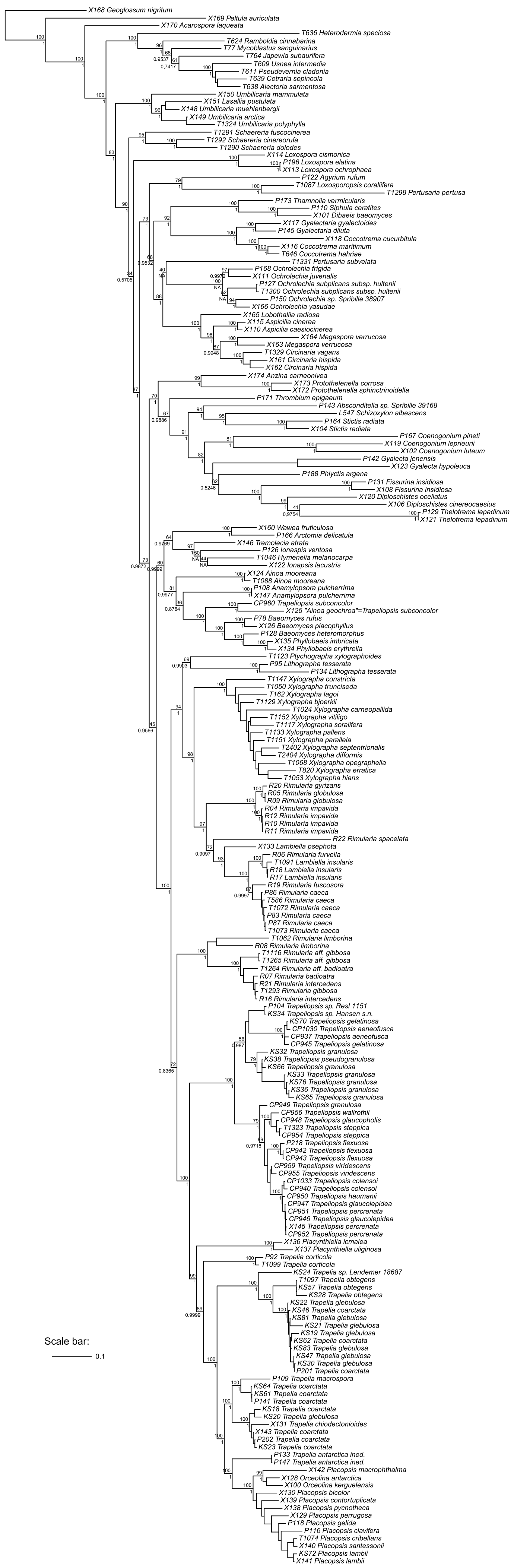

Online Resource 8

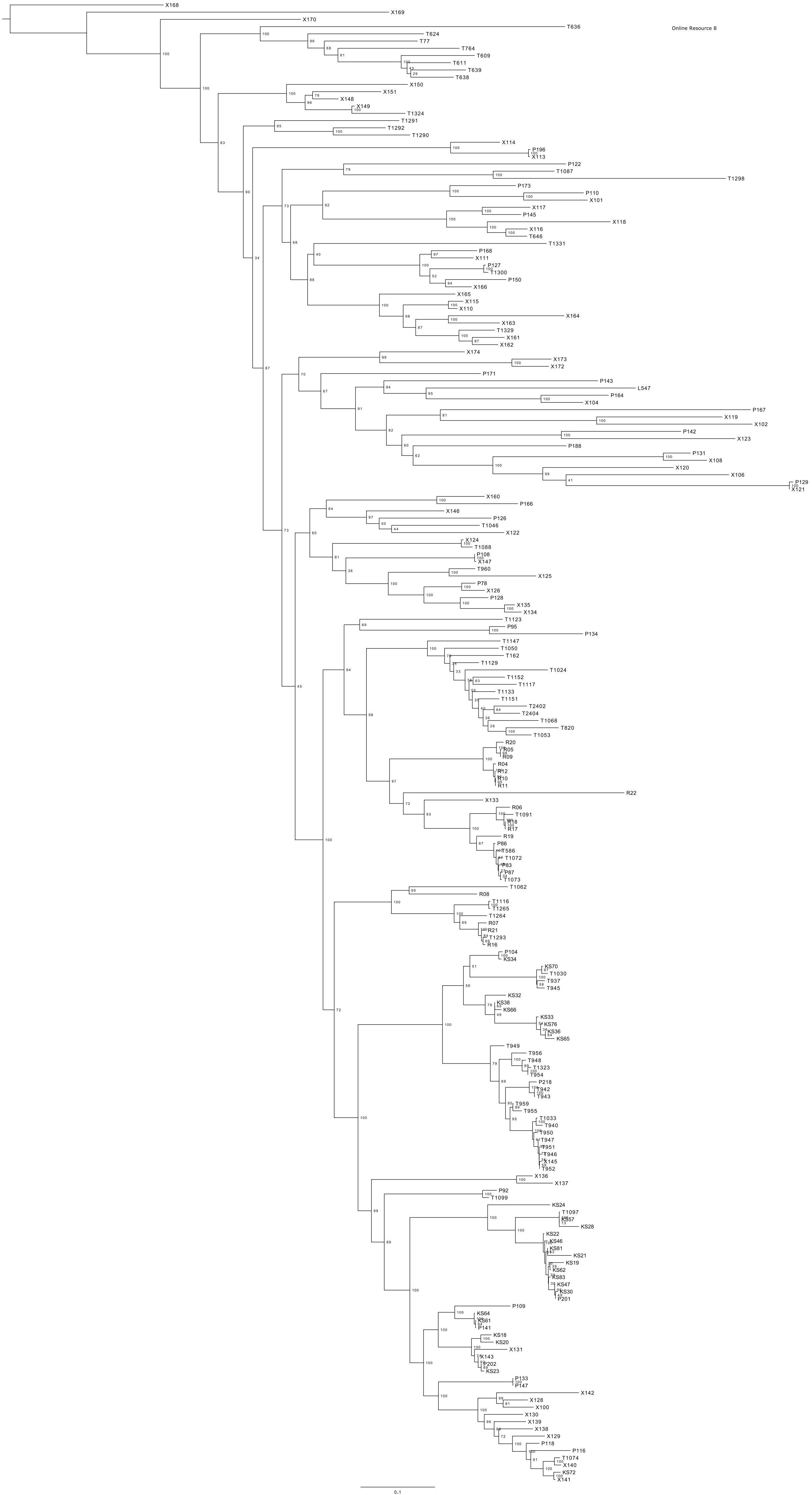

Online Resource 9

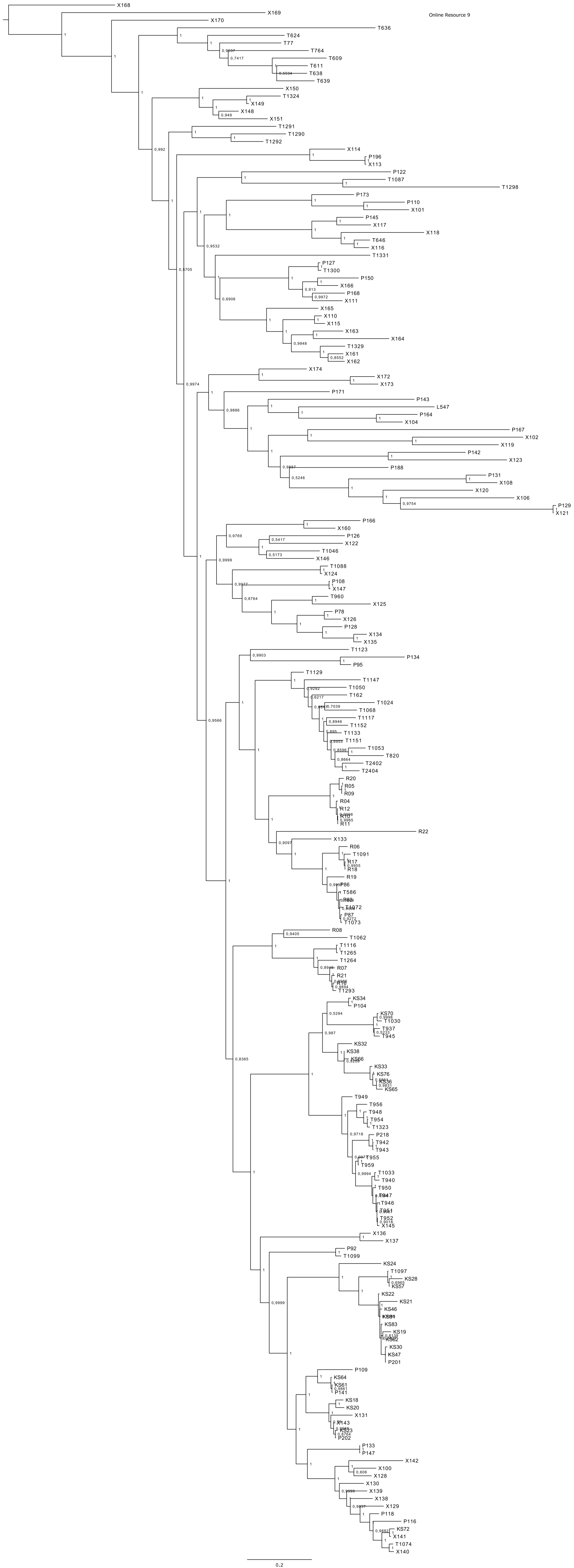

Supplement: Supplementary file 1 — (PDF 5590 kb) [file 13225_2015_332_MOESM1_ESM.pdf]
